# Expression of Exogenous *GFP*-*CesA6* in Tobacco Enhances Cell Wall Biosynthesis and Biomass Production

**DOI:** 10.3390/biology11081139

**Published:** 2022-07-29

**Authors:** Monica De Caroli, Patrizia Rampino, Gabriele Pecatelli, Chiara Roberta Girelli, Francesco Paolo Fanizzi, Gabriella Piro, Marcello S. Lenucci

**Affiliations:** Department of Biological and Environmental Sciences and Technologies, University of Salento, 73100 Lecce, Italy; patrizia.rampino@unisalento.it (P.R.); gabriele.pecatelli@unisalento.it (G.P.); chiara.girelli@unisalento.it (C.R.G.); fp.fanizzi@unisalento.it (F.P.F.); marcello.lenucci@unisalento.it (M.S.L.)

**Keywords:** cell wall, cellulose synthase complex, *AtCesA6*, *Nicotiana tabacum*, cellulose, matrix polysaccharides, plant growth, primary and secondary *CesAs*

## Abstract

**Simple Summary:**

Cellulose is synthesized at the plasma membrane by an enzymatic complex constituted by different cellulose synthase (CesA) proteins. The overexpression of *CesA* genes has been assessed for increasing cellulose biosynthesis and plant biomass. In this study, we analyzed transgenic tobacco plants (F_3_1 line), stably expressing the *Arabidopsis* CesA6 fused to GFP, for possible variations in the cellulose biosynthesis. We found that F_3_1 plants were bigger than the wild-type (wt), showing significant increases of stem height, root length, and leaf area. They bloomed about 3 weeks earlier and yielded more flowers and seeds than wt. In the F_3_1 leaves, the expression of the exogenous *GFP*-*CesA6* prompted the overexpression of all *CesAs* involved in the synthesis of primary cell wall cellulose and of other proteins responsible for plant cell wall building and remodeling. Instead, secondary cell wall *CesAs* were not affected. In the F_3_1 stem, showing a 3.3-fold increase of the secondary xylem thickness, both primary and secondary *CesAs* expression was differentially modulated. Significantly, the amounts of cellulose and matrix polysaccharides increased in the transformed seedlings. The results evidence the potentiality to overexpress primary *CesAs* in tobacco for biomass production increase.

**Abstract:**

Improved cellulose biosynthesis and plant biomass represent important economic targets for several biotechnological applications including bioenergy and biofuel production. The attempts to increase the biosynthesis of cellulose by overexpressing CesAs proteins, components of the cellulose synthase complex, has not always produced consistent results. Analyses of morphological and molecular data and of the chemical composition of cell walls showed that tobacco plants (F_3_1 line), stably expressing the *Arabidopsis* CesA6 fused to GFP, exhibits a “giant” phenotype with no apparent other morphological aberrations. In the F_3_1 line, all evaluated growth parameters, such as stem and root length, leaf size, and lignified secondary xylem, were significantly higher than in wt. Furthermore, F_3_1 line exhibited increased flower and seed number, and an advance of about 20 days in the anthesis. In the leaves of F_3_1 seedlings, the expression of primary *CesAs* (*NtCesA1*, *NtCesA3*, and *NtCesA6*) was enhanced, as well as of proteins involved in the biosynthesis of non-cellulosic polysaccharides (xyloglucans and galacturonans, *NtXyl4*, *NtGal10*), cell wall remodeling (*NtExp11* and XTHs), and cell expansion (*NtPIP1.1* and *NtPIP2.7*). While in leaves the expression level of all secondary cell wall *CesAs* (*NtCesA4*, *NtCesA7*, and *NtCesA8*) did not change significantly, both primary and secondary *CesAs* were differentially expressed in the stem. The amount of cellulose and matrix polysaccharides significantly increased in the F_3_1 seedlings with no differences in pectin and hemicellulose glycosyl composition. Our results highlight the potentiality to overexpress primary *CesAs* in tobacco plants to enhance cellulose synthesis and biomass production.

## 1. Introduction

The cell wall is a dynamic polymeric network surrounding all plant cells and organized in layered structures each characterized by specific properties. Plant cell growth occurs simultaneously to the synthesis and deposition of a primary cell wall consisting of cellulose microfibrils embedded in a hydrated matrix of pectins, hemicelluloses, and proteins. This structural organization ensures tensile strength to oppose turgor pressure and the proper plasticity to allow water uptake and cell expansion [1]. Only some specialized cell types of the mechanical (fibers, sclereids) and vascular (xylem vessels) tissues appose a thickened secondary wall to the primary wall when expansion growth ceases. Strength, rigidity, and hydrophobicity are characteristics of the secondary wall, mainly consisting of cellulose, hemicelluloses, and lignin.

Cellulose is the most abundant polysaccharide of the biosphere with more than 10^11^ tons estimated to be synthesized each year [2]. Cellulose consists of 1,4 linked β-glucan linear chains synthesized at the plasma membrane by the cellulose synthase complex (CSC), a multiprotein complex showing a symmetrical organization in six subunit clusters, each constituted by different cellulose synthase (CesA) proteins. The CSC architecture is not clearly defined, yet. Probably, three different CesA isoforms take part to the synthesis of cellulose in the primary or secondary cell wall. In *Arabidopsis*, CesA1, CesA3, and CesA6-like groups (CesA2, CesA5, CesA6, and CesA9) are involved in primary cell wall growth, whereas the deposition of the secondary cell wall cellulose seems to be coordinated by the CesA4, CesA7, and CesA8 isoforms [3,4,5]. For a long time, cellulose microfibril was assumed to contain 36 β-1,4 glucan chains, supporting the CSC model in which each of the six subunits of the rosette contains six CesAs [6,7,8,9]. Accurate analytical techniques, including atomic force microscopy (AFM), nuclear magnetic resonance (NMR), single molecule imaging, living cell confocal microscopy, and the most advanced structural illumination microscopy (SIM), lean towards a structure consisting of 18–24 β-1,4 glucan chains [10,11,12,13]. Consequently, the “hexamer of hexamers” CSC model has been replaced with the “hexamer of trimers” in which each three CesA isoforms involved in the synthesis of primary or secondary wall are likely present in an equal stoichiometric ratio of 1:1:1 [14,15]. Moreover, new insights on the catalytic activity of single CesAs were recently obtained. The heterologous expression in yeast of *Populus tremula* × *tremuloides CesA8* (*PttCesA8*), involved in secondary cell wall formation, was demonstrated to be sufficient to produce cellulose microfibrils in vitro [16]. Similarly, *Physcomitrella patens* CesA5, involved in primary cell wall formation, reconstituted into proteoliposomes, fostered the synthesis and self-assembly of cellulose microfibrils [17]. In addition, alternative 1:1:1 CSC models have been proposed as in the case of aspen (*Populus tremula* L.) secondary cell wall deposition during xylem vessel differentiation, where CesA8, CesA4, and CesA7 stoichiometry was found to shift from 3:2:1 in normal wood to 8:3:1 in tension wood driven by an increase of *CesA8* expression [18]. Therefore, the organization of CesA proteins yet remains an open question and the possibility that it is species- and/or tissue-specific, as suggested by the studies on aspen, open up new perspectives for biotechnological applications, mainly related to increasing cellulose production and engineering of bioenergy crops.

Several studies have been performed to improve cellulose synthesis by increasing the expression levels of a single *CesA* obtaining unsatisfactory results, especially for secondary wall associated *CesAs*. The overexpression of *PtCesA8* in aspen resulted in silencing of both the exogenous insert and its native counterpart, with a massive cellulose reduction in the secondary xylem and consequent alteration of plant growth [19]. A similar silencing response was reported in barley plants individually transformed with primary (*CesA1*, *CesA2*, and *CesA6*) and secondary (*CesA4* and *CesA8*) cell wall *CesA* cDNAs. No increase of the total cellulose content in any of the transgenic barley lines was obtained and, in some lines, a significant decrease was evidenced other than some aberrant phenotypes [20]. Furthermore, in switchgrass (*Panicum virgatum* L.), CesA4 and CesA6 overexpression resulted in a decrease of cellulose content and crystallinity accompanied by increased xylan content [21]. On the contrary, in *Arabidopsis*, the overexpression of primary cell wall CesA2, CesA5, and CesA6 resulted in an improvement of cellulose biosynthesis and plant growth that seem to be related to changes in expression genes mainly related to cell division and expansion [22].

In a previous study [23], five independently transformed tobacco lines expressing the *AtCesA6* gene fused at the *C*-terminus to the GFP fluorescent tag were obtained aimed at studying the internalization pattern of the resulting GFP-CesA6 chimeric protein [23]. The secretion pattern of the chimera GFP-CesA6 was characterized, highlighting that it recycled between plasma membrane and various subcellular compartments [23] following the CesA recycling pattern that underlies the dynamic remodeling of the cellulose synthase complex [3,24,25]. In the present study, we analyzed one of the stably transformed tobacco lines for possible variations in the synthesis of cellulose. Through different experimental approaches, we show that the expression of the exogenous *GFP-CesA6* affects the biosynthetic machinery involved in the synthesis and remodeling of cell wall, causing an increase of cellulose and matrix polysaccharides, secondary xylem, and plant biomass.

## 2. Materials and Methods

### 2.1. Plants Material and Growth Conditions

Stable transgenic tobacco plants expressing the *GFP-CesA6* construct of the fourth generation, F_3_1 line [23], have been used in this study. *Nicotiana tabacum* cv SR1 (wt) and F_3_1 tobacco seeds were germinated in a growth chamber (temperature 22 °C, humidity 60%, light intensity 25 µE/m^2^/s, photoperiod 16/8 h day/night) on solid MS (Murashige & Skoog, Sigma-Aldrich, St. Louis, MO, USA) medium and transplanted to the soil after two months.

### 2.2. Microscopic Observations and Chlorophyll and Carotenoid Content

Morphometric parameters were measured using ImageJ 1.53e from seed images acquired on a stereomicroscope (Stemi 508, Zeiss, Germany) and on leaves scanned with an EPSON XP-255 scanner. The epidermal pavement cell area was analyzed following the method of Li et al. [26] with some modifications. Epidermal peels were taken from the abaxial lamina of the most expanded leaves of 2-month-old wt and F_3_1 tobacco seedlings and gently pressed onto the slides. Observations were performed under a LSM710 microscope (Zeiss, Germany) at 40× magnification. The area value was measured using the Graphic tool of the Zen2012 Black Edition program.

For stem anatomy observations, the first internode of 2-month-old wt and F_3_1 seedlings was fixed overnight in cold (4 °C) FAA solution (Ethanol 50%, Glacial acetic acid 0.5%, Formaldehyde [37–40%] 10%, water up to the final volume) and then embedded in paraffin. Thin cross-sections (10 µm) were cut with a microtome (Leica RM2155) and picked up on a slide. Clearing procedure, fuchsin A (0.2%) and calcofluor white (0.1%) staining were performed as described by Ursache et al. [27]. Fuchsin A was detected at 561 nm excitation and acquired at 600–650 nm; calcofluor white was detected at 405 nm excitation and acquired at 425–475 nm.

The spectrophotometric determination of leaf content of chlorophylls (a, b, and total) and total carotenoids was carried out according to the Lichtenthaler and Buschmann (2001) method [28] and reported as nmol/g fresh weight (fw).

### 2.3. RNA Extraction and RT-qPCR Analysis

Total RNA was isolated from 2-month-old wt and F_3_1 leaves and stems as previously reported [29]. RT-qPCR analysis was performed using SYBR Green fluorescent detection in a CFX96 Real-Time System Cycler (Bio-Rad, Hercules, CA, USA) with three biological and three technical replicates per sample. The primer sequences used are reported in Table S1. The PCR program was as follows: 10 min at 95 °C; 50 cycles of 15 s at 95 °C; 20 s at 60 °C; and an increment of 0.5 °C every 0.5 s from 65 °C to 95 °C. The specificity of PCR products was checked in a melting-curve test. The *Nicotiana tabacum* Elongation factor *EF-1α* (*EF-1α*, AF120093), *L25* ribosomal protein (*L25*, L18908), and Ubiquitin-conjugating enzyme E2 (*Ntubc2*, AB026056) genes were tested as reference genes [30]. In wt and F_3_1 samples, all genes had a variation coefficient below 0.1, according to Czechowski et al. [31]. The *EF-1α* gene was chosen as the reference gene because it showed the lowest variation coefficient among the genes (Table S2). Differences in gene expression between transformed and wt samples were considered significant when the expression was at least doubled (greater than or equal to two-fold upregulation) or halved (less than or equal to two-fold downregulation), according to Chen et al. [32].

### 2.4. Leaf Proteins Fractionations and Immunoblotting Analyses

Three wt and F_3_1 leaves were finely powdered in a pre-cooled mortar with liquid nitrogen and 100 mg of the powder were suspended in the homogenization buffer (40 mM Hepes-NaOH buffer, pH 7.5, 10 mM imidazole, 1 mM benzamidine, 5 mM 6-aminohexanoic acid, 10 mM dithiothreitol and 1 mM phenyl methyl sulfonyl fluoride) and centrifuged at 800× *g* (Allegra 2IR, Beckman, Brea, CA, USA) for 10 min at 4 °C. The resulting pellet, consisting of cell walls and cell debris, was sequentially washed and extracted to isolate the cell wall protein fraction (CW) according to De Caroli et al. [29]. The 800 g supernatant, containing the protein intracellular fraction, including soluble cargo and intrinsic membrane proteins, was layered on a Ficoll (40%) pad and ultracentrifuged at 100,000× *g* for 2 h at 4 °C (Optima XPN-1000, Beckman). The proteins in the formed interface were collected and delipidated to obtain the intrinsic membrane proteins fraction (MEM); the supernatant was concentrated by filtration on Centricon Plus 20 (Amicon, Merck KGaA, Darmstadt, Germany) to obtain the soluble cargo proteins (SOL) [29]. Protein concentration was determined as described by Bradford [33]. An identical amount of SOL, MEM, and CW protein fractions (10 µg per line) was subjected to SDS-PAGE and Western blotting analysis. Anti-GFP (1:5000 *v*/*v*) (Molecular Probes, now ThermoFisher Scientific, Waltham, MA, USA) and anti-XTH (Agrisera, Vännäs, Sweden) were used in TBS + 1% skimmed-milk powder. All primary antibodies were combined with anti-rabbit secondary antibodies coupled to peroxidase (1:10,000 *v*/*v*) (Sigma-Aldrich, St. Louis, MO, USA). Chemiluminescence reaction occurred using Clarity or Clarity Max (Biorad, Hercules, CA, USA) and the signal was captured by ChemiDoc MP Imaging Systems (Biorad). Densitometric analysis was performed using Image Lab software 6.0.1 (Biorad).

### 2.5. Cell Wall Isolation, Polysaccharide Fractionation and Analyses

Cell walls were isolated, purified (delipidated, destarched, and deproteinated), and essiccated as described by Leucci et al. [34] starting from 10 g fw aliquots of wt and F_3_1 leaves from 2-month-old wt and F_3_1 seedlings. The chemical solubilization of cell wall polysaccharides was carried out according to Lenucci et al. [35]. Aliquots (100 mg) of the purified cell walls were sequentially extracted with (a) 50 mM cyclohexane–trans-1,2-diamine-N,N,N′,N′ tetraacetate (CDTA, Na salt, 10 mL), pH 6.5, at 20 °C for 6 h with shaking (CDTA-1); (b) 50 mM CDTA (10 mL), pH 6.5, at 20 °C for 2 h with shaking (CDTA-2); (c) 50 mM Na_2_CO_3_ + 20 mM NaBH_4_ (10 mL) at 1.0 °C for 16 h with shaking (Na_2_CO_3_-1); (d) 50 mM Na_2_CO_3_ + 20 mM NaBH_4_ (10 mL) at 20 °C for 4 h (Na_2_CO_3_-2); (e) 0.5 M KOH + 10 mM NaBH_4_ (10 mL) at 1.0 °C for 3 h, under nitrogen; (f) 1 M KOH + 10 mM NaBH_4_ (10 mL) at 1 °C for 6 h, under nitrogen; and (g) 4 M KOH + 10 mM NaBH_4_ (10 mL) at 20 °C for 24 h, under nitrogen. At the end of the sequential extractions, an insoluble residue, usually considered α-cellulose, remained. After each extraction, the soluble polymers were separated from the insoluble residue by centrifugation (5000× *g*, 10 min in a Biofuge 15 Heraeus Sepatech centrifuge). The combined alkali extracts and the insoluble residue were acidified to pH 5.0 with glacial acetic acid. The CDTA-1, CDTA-2, Na_2_CO_3_-1, and Na_2_CO_3_-2 extracts were combined and referred to as CDTA + Na_2_CO_3_ extract. Similarly, the 0.5, 1.0, and 4.0 M KOH extracts were combined and referred to as KOH extract. The CDTA + Na_2_CO_3_ and KOH extracts as well as the insoluble residue were dialyzed exhaustively in the presence of 0.05% chlorbutol (with SnakeSkin Pleated Dialysis Tubing, Pierce; Mr cutoff 10,000) against several changes of distilled water for 7 days at 5.0 °C in a cold room, freeze-dried, and accurately weighted. Aliquots (2 mg) of the freeze-dried CDTA + Na_2_CO_3_ and KOH extracts were subjected to acid hydrolysis with 2N trifluoroacetic acid (TFA), at 121 °C for 60 min. For CDTA + Na_2_CO_3_, a pretreatment with 90% formic acid at 100 °C for 6 h was required to improve the release of sugar residues from pectins. The hydrolyzates were centrifuged at 10,000× *g* and the supernatants were taken to dryness in a SpeedVac concentrator (Savant). The amount of cellulose present in the insoluble residue was quantified by the Updegraff [36] method.

To perform ^1^H NMR analysis and spectra acquisition and processing, appropriate aliquots of the dry hydrolyzates (four independent replicas) of wt and F_3_1 transgenic line for both pectin and hemicellulose extracts were processed for ^1^H NMR analysis. For each sample, 600 μL of deuterium oxide (D_2_O) containing 0.05% *w/v* TSP-d4 (sodium salt of trimethylsilyl propionic acid) as a chemical shift reference, δ = 0 ppm, was added to the lyophilized plant material. The content was mixed thoroughly with a vortex mixer at room temperature for 1 min and then filled in a 5 mm NMR tube. All measurements were performed at 300 K on a Bruker Avance III 600 Ascend NMR spectrometer (Bruker, Ettlingen, Germany), operating at 600.13 MHz for ^1^H observation, equipped with a TCI cryoprobe incorporating a *z* axis gradient coil and automatic tuning matching (ATM). For each sample, a 1D sequence with pre-saturation and composite pulse for selection (zgcppr Bruker standard pulse sequence) was acquired, with 128 scans, 16 dummy scans, 5 s relaxation delay, size of fid of 64 K data points, a spectral width of 12,019.230 Hz (20.0276 ppm), and an acquisition time of 2.73 s. The resulting FIDs were multiplied by an exponential weighting function corresponding to a line broadening of 0.3 Hz before Fourier transformation, automated phasing, and baseline correction. Metabolite identifications were based on ^1^H and ^13^C assignment by 1D and 2D omo and heteronuclear experiments (2D ^1^H Jres, ^1^H COSY, ^1^H-^13^C HSQC, and HMBC) and by comparison with the literature data [37,38,39,40,41,42]. NMR data processing was performed by using TopSpin 3.6.1 (Bruker, Biospin, Milano, Italy). All spectra were referenced to the TSP signal (0.00 ppm). The change in the composition of selected components, identified by NMR, between wt and F_3_1 transgenic line hemicelluloses and pectins was determined by analyzing the integrals of selected distinctive unbiased NMR signals after spectra processing, using TSP for chemical shift calibration and quantification [43], after normalization to the amount of hydrolyzate used in each analysis. Differences in the metabolite content were represented as log2foldchange of the calculated average intensities of the corresponding selected signals [44,45].

A TLC analysis was also carried out to visualize the glycosidic residues from the pectin and hemicellulose extracts of wt and transformed leaves. The dried hydrolyzates were solubilized in pyridine and appropriate aliquots (typically 10 µL) deposited on a 20 cm × 20 cm TLC plate (Merck KGaA, Darmstadt, Germany) together with a standard solution containing 5µg/mL of rhamnose, arabinose, galactose, glucose, xylose, mannose, and galacturonic acid. The chromatographic run and the sugar staining were performed according to the method reported by O’Rourke et al. [46]. The ride lasted three hours using the following mobile phase: ethyl acetate:pyridine:acetic acid:water (6:3:1:1, *v*/*v*). The sugars were visualized by immersing the plate in a 0.5% thymol mixture in ethanol:sulfuric acid (19: 1 *v*/*v*) followed by incubation in a pre-heated oven at 105 °C for 5 min.

### 2.6. Protoplast Preparation, Radioactive Labeling and Assay of De Novo Synthesized Cell Wall Polysaccharides

The de novo synthesis of cell wall polysaccharides was assessed on leaf tobacco protoplasts prepared from 2-month-old wt and F_3_1 leaves as described by De Caroli et al. [47]. Freshly prepared protoplasts were incubated in 1 mL of K3M (K3 solution in which sucrose was replaced by mannitol) medium (26 h) in the presence of 430 kBq of D-[U-^14^C]glucose (Amersham Biosciences, Amersham, UK) for the last 8 h of incubation. At the end of the incubation period, the protoplasts were treated as reported in Leucci et al. [48]. Furthermore, to remove the possible presence of callose, the cell walls were incubated with 1,3-β-endoglucanase (barley, Megazyme, Wicklow, Ireland) at 40 °C for 18 h and the residual material was centrifuged at 800× *g* for 10 min at 4 °C to collect purified cell walls. The quantification of newly synthesized matrix polysaccharides and cellulose was performed after nitric acid/acetic acid hydrolysis (Updegraff, [36]) of the protoplasts’ regenerating cell walls and counting for radioactivity of both the hydrolyzates (matrix polysaccharides) and the acid resistant material (cellulose).

### 2.7. Statistical Analyses

Statistical analyses were based on the *t*-Student test. Statistical comparisons were performed using SigmaStat software, version 11.0 (Systat Software Inc., Chicago, IL, USA).

## 3. Results

### 3.1. Expression of the Exogenous GFP-CesA6 Construct in Tobacco Plants

**Figure 1:**
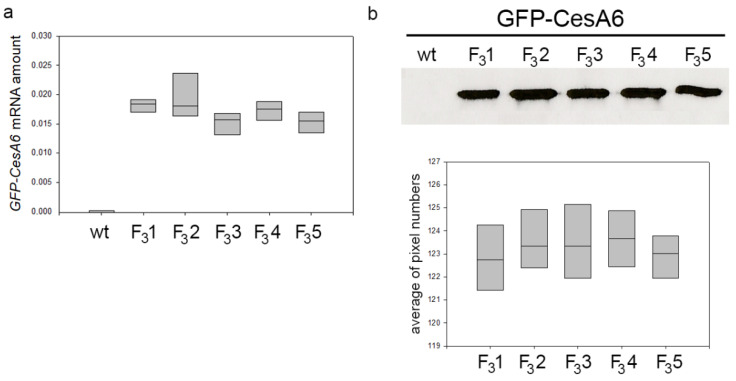
Amount of *GFP-CesA6* in five transformed tobacco lines (F_3_1, F_3_2, F_3_3, F_3_4, and F_3_5). (**a**) RT-qPCR analyses of *AtCesA6*; amplification output values are expressed as 2-∆Cq ± SD for *AtCesA6* mRNA in wt and transformed lines and are considered as proportional to the amount of mRNA target according to Schmittgen and Livak [49]. (**b**) Western blot analyses of membrane protein fractions in leaves of 2-month-old wt and transformed tobacco seedlings stably expressing GFP-CesA6; bands were detected by anti-GFP. The results of three independent biological and three technical replicates are presented with box plots.

At the same growth stage, the five transformed lines of tobacco expressing the exogenous *GFP*-*CesA6* gene were bigger than wt, a character clearly evident already a few weeks after germination. When observed at the confocal microscope, all lines showed similar fluorescence intensity indicating no differences in the expression of *GFP*-*CesA6* [23]. Confocal observations were here confirmed through RT-qPCR analyses revealing comparable expression levels of *AtCesA6* gene in all transformed lines (Figure 1a). Western blot of the membrane protein fractions purified from the leaves of 2-month-old transformed seedlings showed the presence of bands with similar protein level in the five lines (Figure 1b and Figure S1). For subsequent investigations aimed at studying the potential effects of the construct expression on plant growth and cellulose synthesis, among the five lines, we chose the transgenic line F_3_1 as representative of the fourth generation.

### 3.2. Morphometric and Growth Parameters of F_3_1 Seeds and Seedlings

**Figure 2:**
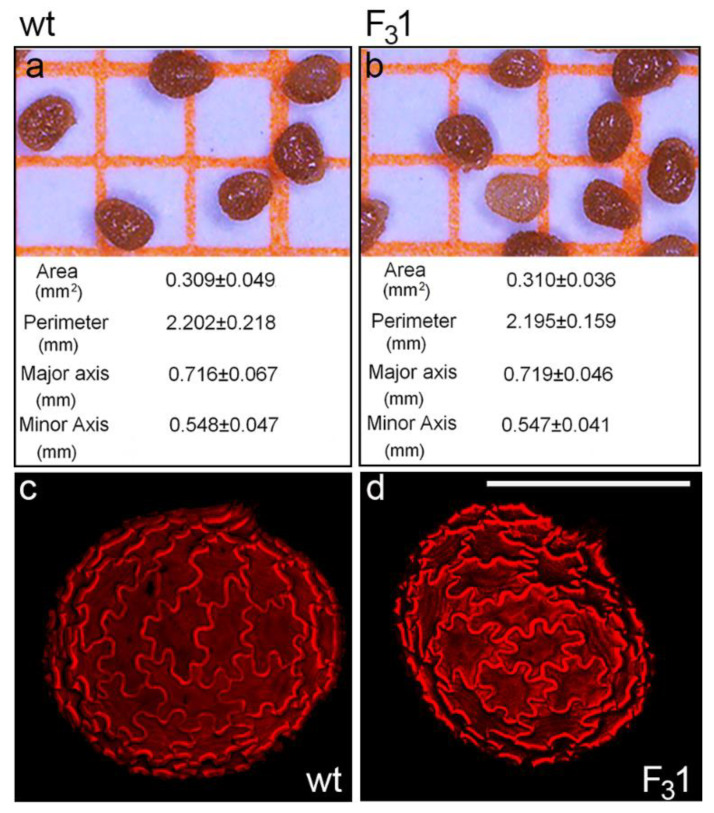
Wt and F_3_1 tobacco seeds. (**a**,**b**) Stereomicroscope images and average values of morphometric parameters of wt and F_3_1 tobacco seeds; the data are the mean ± SD of 100 measurements on wt and F_3_1 seeds. (**c**,**d**) Confocal microscope images of wt and F_3_1 seeds evidence the cell wall autofluorescence of jigsaw-puzzle-shaped pavement cells. Bar scale = 500 µm.

No morphological or morphometric differences were observed between wt and F_3_1 mature dry seeds by stereomicroscope and confocal microscopy. In both cases, seeds appeared oblong-obovate with an eccentric hilum and an average area, perimeter, major and minor axis length of about 0.31 mm^2^, 2.20 mm, 0.72 mm, and 0.55 mm, respectively (Figure 2a,b). The outer integument of the seed coat was brown pigmented with a striate texture (Figure 2a,b) and constituted of normally expanded jigsaw-puzzle-shaped cells, as clearly visualized by the autofluorescence of their thickened cell walls (Figure 2c,d).

**Figure 3:**
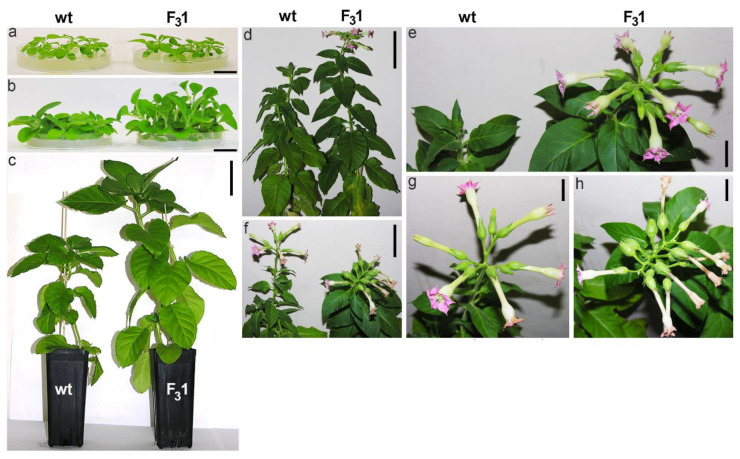
Tobacco wt and F_3_1 plant phenotypes at different stages of growth. (**a**) 1-month-old tobacco seedlings; (**b**) 2-month-old tobacco seedlings; (**c**) 5-month-old tobacco plants; (**d**) tobacco plant phenotype at the flowering stage; (**e**) enlarged detail of (**d**); (**f**) flowers; (**g**,**h**) enlarged detail of (**f**). Bar scale: 2 cm (**a**,**b**), 10 cm (**c**), 7 cm (**d**,**f**), 2 cm (**e**,**g**,**h**).

Although transformation did not affect neither the rate (>98%) nor the seed germination time (4 days after sowing), a slight increase in size was macroscopically noticeable in 30-day-old F_3_1 seedlings compared to wt, a difference which became clearly apparent 2 months after sowing and was remarkable in the 5-month-old plants (Figure 3a–c).

**Figure 4:**
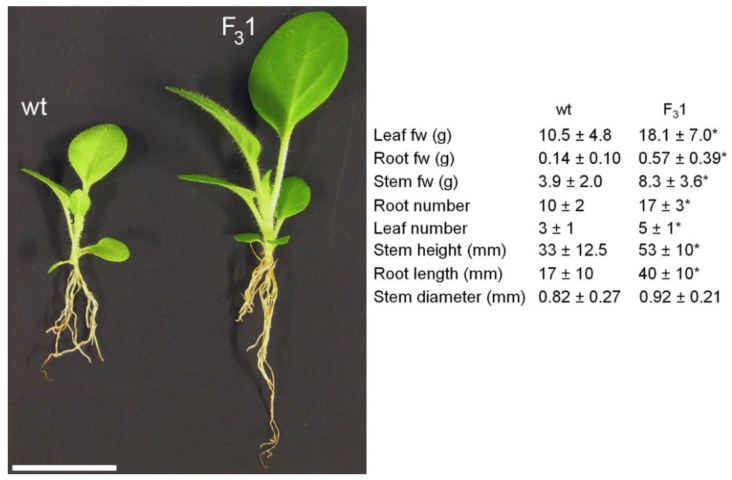
Morphometric parameters of 2-month-old wt and F_3_1 seedlings. The data are the mean ± SD of 50 samples examined for each line. Asterisk indicates significant differences (*p* < 0.05; *), according to the *t*-Student test, between the two samples. fw: fresh weight. Bar scale = 2 cm.

Adult F_3_1 plants bloomed about 15–20 days earlier than wt, showing an increased number of flowers and seeds (Figure 3d–h, Table 1). A significant (*p* < 0.05) increase in the average of both stem height (from the root collar to the shoot apex) and root length of about 61% and 135%, respectively, was evidenced in the 2-month-old F_3_1 seedlings compared to wt, as well as in the number of true leaves (67%) and roots (70%), and in leaf, root, and stem fresh weights (fw, 72%, 307%, and 113%, respectively) (Figure 4).

**Figure 5:**
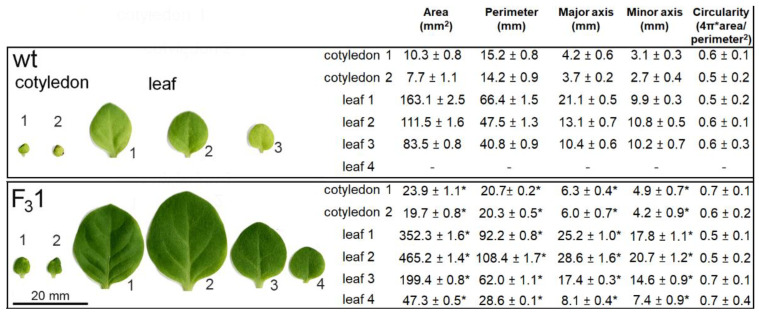
Morphometric parameters of wt and F_3_1 leaves and cotyledons of 2-month-old seedlings. An amount of 30 leaves for each sample were examined for each line. The data represent the mean ± SD. Asterisk indicates significant differences (*p* < 0.05; *), according to the *t*-Student test, between the two samples.

Moreover, when the morphometric parameters (area, perimeter, major and minor axes, and circularity) of embryonal (cotyledons) and true leaves (progressively numbered according to their emergence order) were measured, all traits, except circularity, appeared significantly increased in the 2-month-old F_3_1 seedlings with respect to wt (Figure 5).

**Figure 6:**
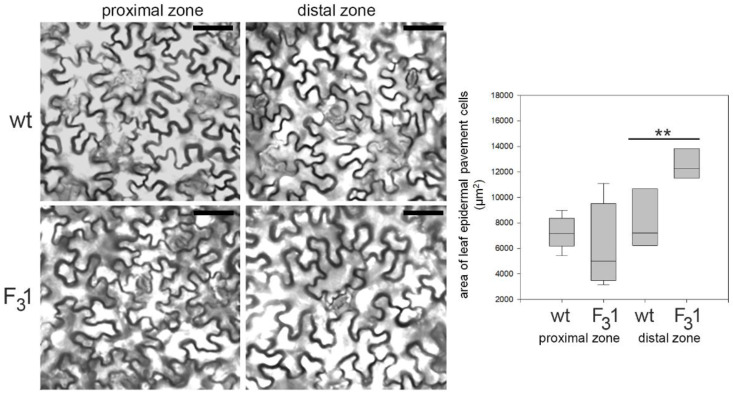
Representative transmitted light confocal images of wt and F_3_1 leaf epidermal pavement cells of abaxial lamina and quantitative evaluation of the cell area in wt and F_3_1 line. The data are reported with box plot graph. Asterisks indicate highly significant differences (*p* < 0.001; **), according to the *t*-Student test, between the two samples.

Remarkably, the two cotyledonary leaves, emerging during seed germination, also appeared more than twice as large in the transgenic line than in wt. In the leaf numbered 2 (Figure 5), the size and shape of wt and F_3_1 abaxial epidermal cells were compared with the corresponding equal areas of proximal and distal zone. In the proximal zone, the cells showed the same pattern and approximately constant average cell area; in the distal zone, whereas the shape does not change, the area of the F_3_1 epidermal cells significantly increased by expansion growth with respect to the wt (Figure 6).

On a fw basis, the leaves of 2-month-old F_3_1 seedlings showed a slightly reduced content of chlorophyll a (Chl a, 123.9 nmol/g fw) and total chlorophylls (Chl (a+b), 155.6 nmol/g fw) than wt (143.1 and 181.9 nmol/g fw, respectively). Instead, no significant variations were found in the levels of chlorophyll b (Chl b) and total carotenoids (Table 2). Furthermore, the ratio Chl a:Chl b slightly increased in F_3_1 (3.9) with respect to the wt (3.7), while the ratio between Chl (a+b) and total carotenoids (Car) revealed a clear decrease in the F_3_1 (2.86) than wt (3.53) leaves.

Thus, apart from the “giant” phenotype and larger fresh biomass production, no aberrant morphological traits (i.e., curled or asymmetrical leaves, necrotic or differently pigmented areas, defective flower morphology) were observed in F_3_1 plants.

### 3.3. Exogenous GFP-CesA6 Increases the Expression of Endogenous Primary CesAs and of Other Genes Involved in the Biosynthesis and Remodeling of Cell Wall Polymers in Tobacco Leaves

To investigate whether the enhanced growth of F_3_1 seedling was supported by a general overexpression of cell wall synthesis-, deposition-, and/or remodeling-related genes, we performed a RT-qPCR on RNA samples isolated from the leaves of 2-month-old transformed and wt seedlings. In particular, we examined the expression levels of the endogenous *CesAs* involved in cellulose synthesis of primary (*NtCesA1*, *NtCesA3*, and *NtCesA6*) and secondary (*NtCesA4*, *NtCesA7*, and *NtCesA8*) cell walls, as well as of other genes pivotal for matrix polysaccharide (pectins and hemicelluloses) metabolism and cell wall expansive growth (namely *NtXyl4*, *NtGal10*, *NtExp11*, *NtPIP1.1*, and *NtPIP2.7*). In particular, *NtXyl4* encodes for an UDP-glucose:xyloglucan 1,4-β-D-glucosyltransferase putatively involved in backbone elongation of xyloglucans; the product of *NtGal10* is a protein with putative galacturonosyltransferase activity catalyzing pectin biosynthesis, while *NtExp11* codes for an expansin-A11 causing loosening and extension of plant cell walls disrupting the non-covalent bonding between cellulose microfibrils and matrix glucans. *NtPIP1.1* and *NtPIP2.7* code for two plasma membrane aquaporins involved in roles typically reported for PIPs across plants species, including leaf cell expansion [50].

**Figure 7:**
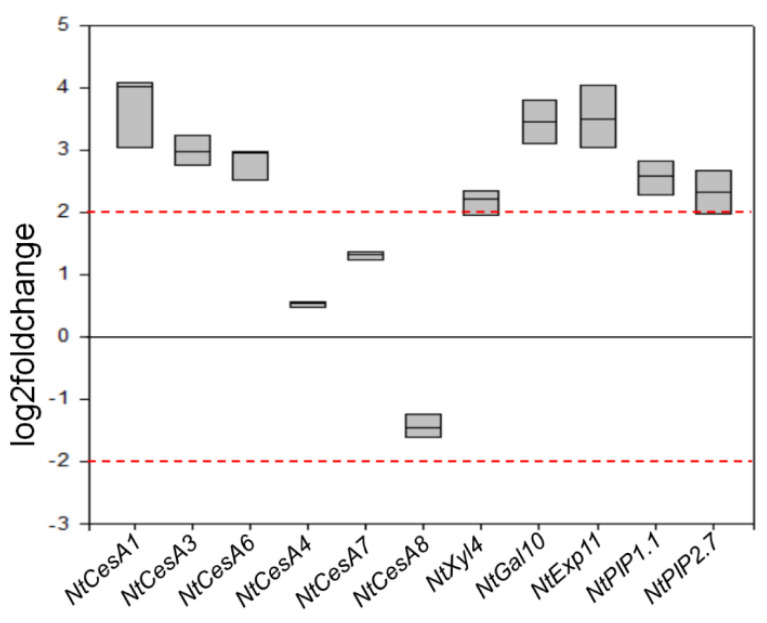
RT-qPCR analyses of *NtCesA1*, *NtCesA3*, *NtCesA6*, *NtCesA4*, *NtCesA7*, *NtCesA8*, *NtXyl4*, *NtGal10*, *NtExp11*, *NtPIP1.1*, and *NtPIP2.7* genes in leaves of 2-month-old wt and F_3_1 tobacco seedlings. The gene expression is reported as transcript inhibition level in F_3_1 leaves (log2 of fold change) with respect to wt. The results of three independent biological and three technical replicates are presented with box plots. The horizontal red dashed lines indicate the significant threshold of log2foldchange.

Compared to wt, F_3_1 seedlings showed an overexpression of *NtCesA1* (log2Foldchange between 3 and 4), *NtCesA3*, and *NtCesA6* (log2Foldchange between 2 and 3) but not of *NtCesA4*, *NtCesA7*, and *NtCesA8*. *NtGal10* and *NtExp11* were also both strongly upregulated (log2Foldchange between 3 and 4), while the expression level of *NtXyl4* was only slightly above the typical significance threshold of 2 for log2Foldchange. *NtPIP1.1* and *NtPIP2.7* were also upregulated with a log2Foldchange between 2 and 3 (Figure 7).

**Figure 8:**
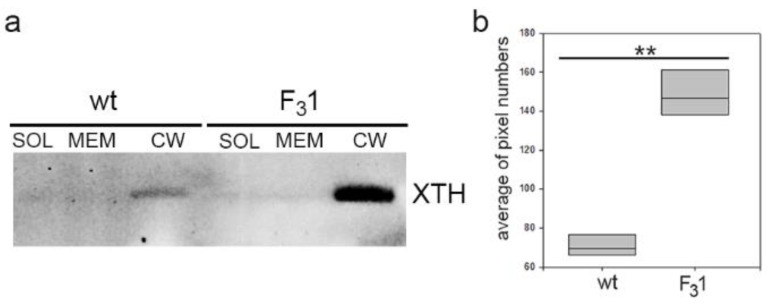
Western blot of soluble (SOL), membrane (MEM), and cell wall (CW) proteins of wt and F_3_1 tobacco leaves of 2-month-old seedlings. (**a**) XTH abundance in the cell wall fraction protein of wt and F_3_1 lines; (**b**) quantification of XTH abundance, reported as average of pixel numbers represented with box plots. Immunolocalization was carried out with an anti-XTH serum. Asterisks indicate highly significant differences (*p* < 0.001; **), according to the *t*-Student test, between the two samples.

Additionally, Western blot analyses of protein extracts from purified soluble, membrane, and wall fractions isolated from the leaves of 2-month-old tobacco wt and F_3_1 seedlings were performed to get direct indications on the total abundance of the xyloglucan endotransglicosylase/hydrolases (XTHs). XTHs are a class of enzymes with a role in the assembly, growth, and remodeling of plant cell walls catalyzing the molecular grafting between xyloglucans of the hemicellulose-cellulose network [29,51]. Immunoblots with the anti-XTH serum revealed a single protein band present exclusively in the cell wall fraction with an approximate MW of 40 KDa compatible with that of the target proteins and with intensity about 4-fold higher in the transformed line than in wt (Figure 8 and Figure S2).

### 3.4. In the Stem of GFP-CesA6 Transformed Tobacco Seedlings the Secondary Xylem Thickness Was Enhanced

**Figure 9:**
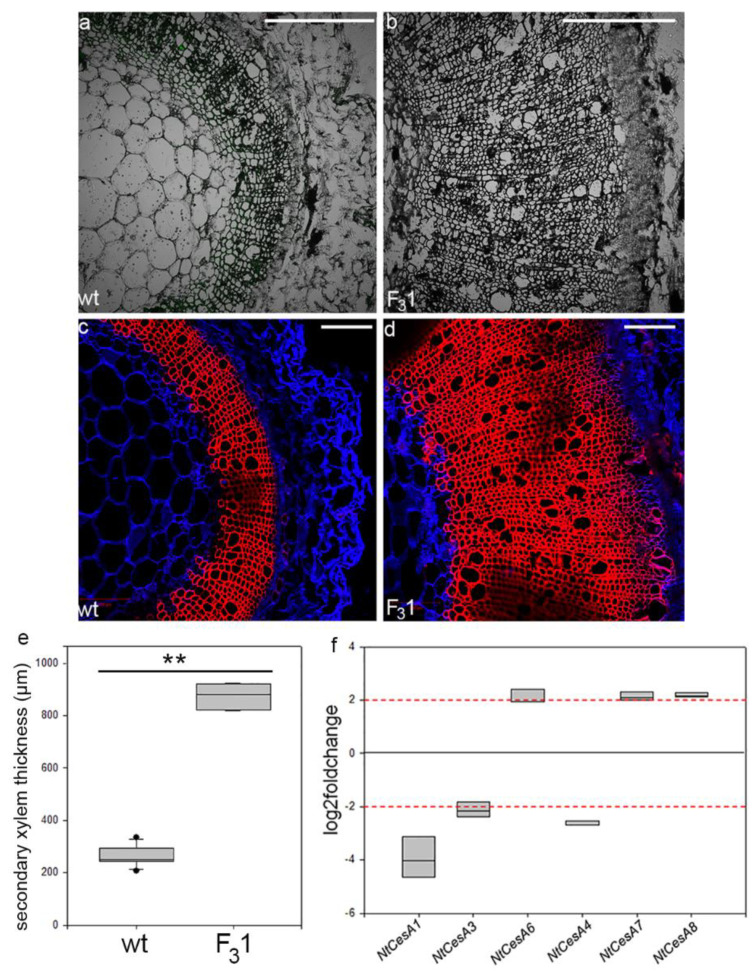
Confocal microscope images of transverse sections of the stem first internode of 2-month-old wt and F_3_1 tobacco seedlings. (**a**,**b**) Transmitted light confocal images; (**c**,**d**) transverse sections stained with fuchsin A (red) and calcofluor white (blue). Bar scale = 500 µm. (**e**) Quantification of secondary xylem in the stem of wt and F_3_1 tobacco seedlings. The measurements are reported with box thickness plot graph. Asterisks indicate highly significant differences (*p* < 0.001; **), according to the *t*-Student test, between the two samples. (**f**) RT-qPCR analyses of *NtCesA1*, *NtCesA3*, *NtCesA6*, *NtCesA4*, *NtCesA7*, and *NtCesA8* genes in the stem of 2-month-old wt and F_3_1 tobacco seedlings. The gene expression is reported as transcript inhibition level in F_3_1 leaves (log2foldchange) with respect to wt. The results of three independent biological and three technical replicates are presented with box plots. The horizontal red dashed lines indicate the significant threshold of log2foldchange.

From the transmitted light confocal observations of transverse sections (10 µm thick) of first internode of 2-month-old seedlings, no anomalies in the histological elements were revealed (Figure 9a,b). The same sections, stained with fuchsin A and calcofluor white (specific for lignin and cellulose, respectively), showed a 3.3-fold increase of the secondary xylem thickness in the F_3_1 line (874 ± 45 µm) compared to wt (262 ± 38 µm) (Figure 9c–e). Gene expression analyses of the stem first internode revealed differentially primary and secondary *CesAs* expression profiles (Figure 9f). While the expression of the primary *NtCesA1* and *NtCesA3* was significantly reduced with respect to the wt (log2Foldchange between −2 and −4), the NtCesA6 expression was increased (log2Foldchange around 2). With regard to the secondary *NtCesAs*, the expression level of *NtCesA8* and *NtCesA7* genes increased (log2Foldchange around 2 for both genes) while *NtCesA4* expression was reduced (log2Foldchange between −2 and −3).

### 3.5. The Expression of the GFP-CesA6 Construct Affects the Relative Amounts of Cell Wall Polysaccharides

To understand if the stable transformation with the *GFP-CesA6* construct was associated with alterations in the levels of cellulose and/or matrix polysaccharides, the cell walls from the leaves of 2-month-old seedlings (wt and F_3_1) were isolated, purified, and sequentially extracted into three fractions enriched in pectins (CDTA + Na_2_CO_3_ extracts), hemicelluloses (0.5–4.0 M KOH extracts), and cellulose (insoluble residue). The dry weight (dw) of the purified cell walls, expressed in mg/g fw, was significantly different (*p* < 0.05) between F_3_1 (17.6 ± 1.3) and wt (7.3 ± 0.6), with up to a 2.4-fold increase (Table 3).

Moreover, the weight of CDTA + Na_2_CO_3_ and KOH extracts obtained from F31, expressed as mg dw/g tissue fw, were slightly higher (10% and 23%, respectively) than those obtained from wt leaves, while the weight of the insoluble residue was increased over 3 times in the transformed line (15.0 ± 2.28 mg/g fw) compared to wt (4.18 ± 0.56). In percentage terms, pectins and hemicelluloses of wt leaves contributed to about 23% and 11% of the total dw of the cell wall, while the remaining 66% consists of the insoluble residue, which turned out to contain about 18.2% of crystalline cellulose as estimated by the Updegraff method [36]. The weight of the insoluble residue from F31 was much higher (84.9%, containing 23.7% crystalline cellulose), at the expense of pectins and hemicelluloses, which contributed, respectively, to 9.9% and 5.2% of the purified cell wall dw.

Significant differences were also detected calculating the percentage of pectins, hemicelluloses, and cellulose relative to their total weight (Table 4). In the walls of wt seedling leaves, pectins represented the prevalent cell wall polysaccharide (44.2%), followed by cellulose (35.2%) and hemicelluloses (20.6%). Transformation, instead, determined a considerable increase in the percentage of cellulose, which, in the F_3_1 leaves, contributes to 61.1% of the total weight of cell wall polysaccharides, followed by pectins (25.6%) and hemicelluloses (13.3%).

**Figure 10:**
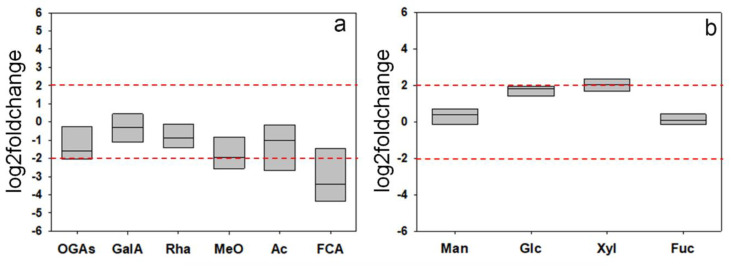
Log2foldchange in the composition of some selected components of the hydrolyzates from CDTA + Na_2_CO_3_ (pectins, (**a**)) and KOH 0.5–4.0 M (hemicelluloses, (**b**)) extracts obtained from the purified cell walls from the leaves of wt and F_3_1 tobacco 2-month-old seedlings. Data of four independent experiments are presented. GalA, galacturonic acid; OGAs, Oligogalacturonides; Rha, rhamnose MeO, Methoxy groups; Ac, Acetyl groups; FCA, ferulic acid; Man, mannose; Glc, glucose; Xyl, xylose; Fuc, fucose. The horizontal red dashed lines indicate the significant threshold of log2foldchange.

For a rough indication of the glycosidic composition of the structural polysaccharides, the CDTA + Na_2_CO_3_ and KOH extracts were subjected to acid hydrolysis, and the resulting products were assayed both by ^1^H-NMR and thin layer chromatography (TLC). Both analytical techniques showed qualitatively similar profiles of the wt and F_3_1 hydrolyzates. The relative concentrations of the various metabolites in the different ^1^H-NMR spectra were calculated by integrating the area of selected distinctive unbiased NMR signal. In particular, signals at 7.14 ppm (ferulic acid), 5.32 ppm (α-galacturonic acid monomers), 5.22 ppm (galacturonic acid oligomers), 5.10 ppm (rhamnose), 4.64 ppm (β-galacturonic acid monomers), 3.30 ppm (methoxy groups), and 2.22 ppm (acetyl groups) were selected and integrated for pectin wt and F_3_1 samples (Figure S3a,b). Moreover, signals at 5.25 ppm (α-fucose), 5.22 ppm (α-glucose, 5.18 ppm (α-xylose), 5.16 ppm (α-mannose), 4.88 ppm (β-mannose), 4.63 ppm (β-glucose), 4.56 ppm (β-xylose), and 4.50 ppm (β-fucose) were selected and integrated for hemicellulose wt and F_3_1 samples (Figure S4). CDTA + NaC_2_O_3_ hydrolyzates revealed the presence of signals attributable to galacturonic acid and rhamnose, as well as of a mixture of oligomeric fragments, confirming the low susceptibility of pectins to acid hydrolysis [52]. The presence of methoxy and acetyl groups, probably esterified to the carboxyl and hydroxyl groups of the C2 and/or C3 of galacturonic acid residues [53], and ferulic groups, known to contribute to the formation of cross-links between the pectic polysaccharides and between them and structural wall proteins [54], were also evidenced. Mannose, glucose, xylose, and fucose were the main glycosyl residues identified in the KOH extracts (Figure S4). The data, expressed as log2foldchange, showed a general trend towards a reduction of the areas of ^1^H-NMR signals of galacturonic acid, rhamnose, oligogalacturonides, methoxy, acetyl, and ferulic groups between F_3_1 and wt lines, although most of these variations (with the exception of ferulic acid) were not significant at the chosen threshold (+2, −2 log2foldchange) (Figure 10a). On the contrary, an upward trend was found for glucose and xylose in the KOH extracts (Figure 10b).

The TLC (Figure S5) of the glycosidic residues deriving from the CDTA + Na_2_CO_3_ extracts obtained from wt and transformed leaves confirmed the presence, in both samples and with no substantial differences, of galacturonic acid and rhamnose, as well as of glucose, xylose, and mannose. Similarly, the hydrolyzates from the extracts in KOH does not show evident differences in the glycosidic composition of the hemicelluloses related to the transformation. In agreement with ^1^H-NMR, the most represented sugars were glucose and xylose, main constituents of the xyloglucans, principal hemicellulose of dicotyledonous primary cell walls. The presence of spots corresponding to arabinose, mannose, and rhamnose residues was also evident.

**Figure 11:**
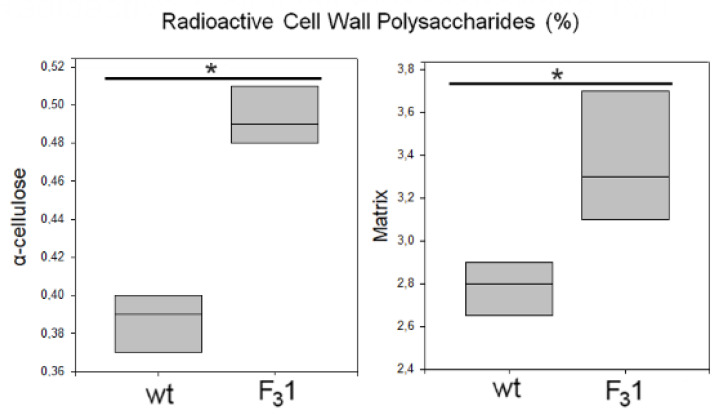
Biosynthesis of cell wall polysaccharides in wt and F_3_1 tobacco leaf protoplasts. Protoplasts were incubated in the presence of 430 kBq of D-(U-^14^C)glucose for 8 h. Radioactive matrix cell-wall polysaccharides and α-cellulose were isolated from wt and F_3_1 tobacco leaf protoplasts. Radioactivity is reported as percentage of the homogenate (approx. 23 kBq for both wt and F_3_1 lines). Data are represented as box plots of three independent experiments. Asterisk indicates significant differences (*p* < 0.05; *), according to the *t*-Student test, between the two samples.

The de novo synthesis of α-cellulose was also followed by radiolabeling of freshly prepared leaf protoplasts from 2-months-old seedlings in the presence of D-[U-^14^C]glucose for 8 h. At the end of the incubation period, the regenerating cell walls were isolated and purified (destarched, deproteinated, and treated with 1,3-β-endoglucanase to remove eventual callose). The radioactivity incorporated into the insoluble residue resulting after acetic acid/nitric acid hydrolysis of the wt and F_3_1 purified cell walls, represented, respectively, 0.39% and 0.49% of the total radioactivity of the homogenate, corresponding to a 25.6% increase of cellulose biosynthesis in the F_3_1 protoplasts (Figure 11).

Significantly, the radioactivity associated to the acetic acid/nitric acid hydrolyzates (matrix polysaccharides) was also increased of approximately 21% in the F_3_1 line, being their amount 2.8% and 3.4% in the wt and in the transformed seedling protoplasts, respectively. These results once again showed the enhanced effect of the insertion of *GFP*-*CesA6* gene on the whole cell wall synthesis and in general on the plant growth.

## 4. Discussion

Cellulose, the main plant cell wall polysaccharide, is used in several industrial applications including paper, textile, food/feed, and bioethanol production [55]. In flowering plants, more than 80% of dry biomass consists of cell wall polymers, mainly polysaccharides, with cellulose accounting for approximately 35–45% of its composition [56]. Increasing cellulose biosynthesis, thus, is of considerable economic and social importance and represents an important goal to improve plant growth, biomass production, and wood quality and quantity. Among other experimental approaches, the overexpression of *CesA* genes has been appraised to increase cellulose production and, in general, to obtain biomass-producing crop plants. Economically important trees (poplar), as well as herbaceous (barley) and model plants (*Arabidopsis*), have been transformed for this purpose with contradicting results. Indeed, genetic manipulation of *CesA* members, particularly those involved in secondary wall biosynthesis, remains difficult to handle yet [19,20,57,58].

In this study, we demonstrate that the transgenic tobacco line F_3_1, expressing the exogenous *GFP-CesA6* construct, shows an increased plant biomass, matrix and cellulose biosynthesis, and a “giant” phenotype, with no apparent other morphological aberrations. Compared to wt, F_3_1 seeds did not show variations neither in morphology nor in size (Figure 2). Moreover, no significant differences were observed in seed germinability and sprout morphology. Changes in growth were visible already one month after sowing and, in a much more relevant way, at later growth stages (Figure 3). The 2-month-old F_3_1 seedlings exhibited higher values of all evaluated growth parameters (Figure 3 and Figure 4), with an approximately 22% and 43% significant increase of stem and root length, respectively, as well as of leaves morphometric parameters. Significantly, aberrant forms were never detected during the development of stems, roots, and leaves, indicating that the F_3_1 line has no morphological or physiological anomalies. An anticipation of the anthesis and an increase of the average flower number per plants, total seed number, and weight (Figure 3, Table 1) also characterized the F_3_1 line.

A relationship between photosynthetic activity and transgenic plant biomass increase was hypothesized in the poplar hybrid clone “Nanline895” (*Populus deltoids* × *Populus euramericana*) overexpressing *CesA2* gene from *Pinus massoniana* under the control of constitutive 35S promoter. The transgenic plants showed increased chlorophyll content, secondary wall thickening, and xylem width, as well as improved cellulose synthesis, plant growth, and biomass accumulation [59]. Differently from transgenic poplar, the leaves of 2-month-old F_3_1 tobacco seedlings showed a slight but significant reduction in the content of chlorophyll a, the main pigment providing energy for oxygenic photosynthesis and emitting high energy electrons into the P680 and P700 photosystems. The amount of chlorophyll b and total carotenoids, both playing a role in light harvesting as accessory pigments, as well as the total content of chlorophylls remained instead unaffected.

The conflicting results are likely related to the different experimental systems investigated, i.e., expression of different genes involved in the biosynthesis of the primary cell wall (*CesA6* and *CesA2* in tobacco and poplar, respectively), use of diverse promoters (the endogenous *CesA6* promoter in tobacco, the strong CaMV 35S constitutive promoter in poplar), and distinct growth habitus of the two species (herbaceous vs. arboreal). Indeed, the parameters related to chlorophylls concentrations significantly differed among plant functional groups (trees < shrubs < herbs) [60]. Moreover, several factors seem to regulate the expression of genes involved in chlorophyll biosynthesis. In *N. benthamiana*, for example, the expression of a DNA virus-encoded protein has been reported to cause structural and functional damages to chloroplasts and to inhibit photosynthesis [61].

The slight increase of Chl a: Chl b and Chl (a+b):Car ratios in the F_3_1 leaves suggests a process of light acclimatization possibly resulting from an indirect effect of transformation. Thus, taller F_3_1 plants, getting closer to the artificial light, activate adaptation and photoprotection mechanism reducing chlorophylls and simultaneously increasing carotenoid amounts [61]. Anyway, Chl a: Chl b ratio of both samples falls within the range of physiological variations, confirming that there is no indication of possible damage in F_3_1 plants [62,63].

Transformation with the exogenous *GFP-CesA6* construct also enhanced the expression of endogenous primary *NtCesAs* (*NtCesA1*, *NtCesA3*, and *NtCesA6*) and of proteins not strictly related to cellulose biosynthesis but, more generally, responsible for plant cell wall building and modification (*NtXyl4*, *NtGal10*, *NtExp11*, *NtPIP1.1*, and *NtPIP2.7*). On the contrary, all cell wall secondary *CesAs* (*NtCesA4*, *NtCesA7*, and *NtCesA8*) were not significantly affected in the leaves of the F_3_1 line, indicating that the two sets of *CesA* genes are not related, at least in this organ. In *Arabidopsis*, the overexpression of *CesA2*, *CesA5*, and *CesA6* was reported to enhance the expression of other primary *CesAs* and of the cellulose-related genes *KORRIGAN2*, *SUS1*, *COBRA*, *CSI1*, and *CSI3* [22]. A simultaneous increase of cell division and expansive growth and of secondary cell wall deposition rate was also revealed, corroborating the idea that primary wall *CesA* engineering could affect plant growth in other ways than simply increasing cellulose biosynthesis. In close agreement with the report of Hu et al. [22], our results show that various components of the cell wall biosynthetic machinery are affected by the insertion of *GFP-CesA6* construct, including those involved in the biosynthesis of non-cellulosic polysaccharides (xyloglucans and galacturonans) and in cell wall remodeling (i.e., *NtExp11* and XTHs).

The expression of the *GFP-CesA6* construct seems to also affect the relative amounts of cell wall polysaccharides. Indeed, a significant 2.4-fold increase of the purified cell wall material obtained per g of fw tobacco leaves was detected in the F_3_1 line. This was mainly attributable to an increase of the insoluble residue following the extraction of pectins and hemicelluloses and of the cellulose contained therein (Table 3). Interestingly, the contribution of total polysaccharides to the dw of the purified cell walls (Table 4) was slightly but significantly lower in the F_3_1 line than in the wt, suggesting that the biosynthesis of non-polysaccharide material may be stimulated by the transformation. Furthermore, the transformed plants had a considerable increase in the percentage of cellulose which, in the leaves, contributes to 61.1% of the total weight of the wall polysaccharides, followed by pectins (25.6%) and hemicelluloses (13.3%); in wt, pectins represent the prevalent polysaccharide (44.2%), followed by cellulose (35.2%) and hemicelluloses (20.2%). Similarly, a higher percentage of cellulose associated with a reduction of monosaccharides involved in the biosynthesis of hemicelluloses and pectins and a giant phenotype was previously reported by Hu et al. [22] in *Arabidopsis* plants overexpressing different *CesA* subunits. No significant difference emerged, instead, in the monosaccharide composition and in the degree of methylation and acetylation of the analyzed fractions which, in both wt and F_3_1 leaves, are compatible with the presence of pectic polysaccharides of the type of homogalacturonans and rhamnogalacturonans I and II, and of hemicelluloses of the type of xyloglucans characteristic of the type I primary wall of dicotyledonous Angiosperms. A significant decrease of polysaccharide feruloylation was also evidenced in the F_3_1 line, even though ferulic acid is reported to be a minor constituent of the tobacco leaf cell walls [64]. In addition, we also found that F_3_1 leaf protoplasts incubated in the presence of radioactive glucose showed an increased amount of cellulose biosynthesis and, significantly, also of matrix polysaccharides. Therefore, our data evidenced a correlation of the increased expression level of the cellulosic and non-cellulosic polysaccharides encoding genes to an actual increase of all the cell wall polysaccharides.

Although in leaves the exogenous expression of *GFP*-*CesA6* had no effect on the expression level of wall secondary *CesAs*, in the stem of 2-month-old F_3_1 seedlings, variations of *CesAs* expression were revealed showing a downregulation of primary *CesA1* and *CesA3* and secondary *CesA8*, and an increased expression of primary *CesA6* and secondary *CesA4*. Furthermore, confocal microscope observations of cross-sections of F_3_1 first internode showed a remarkable increase in the diameter of the secondary lignified xylem with respect to wt, suggesting an organ specificity of the transformation effects. In *Arabidopsis*, all primary CesAs are reported to physically interact with secondary CesAs, both in vitro and in *planta*. The possibility that mixed complexes of primary and secondary CesAs may occur at particular times has been suggested, mainly during the transition between primary and secondary cell wall [65]. Our differential expression of primary and secondary *CesAs* would seem to corroborate this hypothesis.

In *Arabidopsis*, the overexpression of *CesA6*-*like* genes has been suggested to influence both cell expansion and division [22]. Furthermore, it has been reported that several cellulose synthase-like (*Csl*) genes promote cell division and affect cell cycle, including *CslD5* during *Arabidopsis* stomatal lineage development [66] and in meristems [67], *CslD1* in maize [68], *CslDs* (*CslD1*, *CslD2a*, *CslD2b*, *CslD3a*, *CslD5*) in *Dendrobium catenatum* [69], and *CslD4* in rice [70]. The increase of all parameters related to the dimension of F_3_1 stem, leaves, and roots is in line with the above-mentioned results, suggesting that both cell division and cell expansion are increased in the F_3_1 line. Indeed, a stimulus of the vascular cambium meristematic activity is strictly related to the sharp increase of secondary lignified xylem thickness observed in F_3_1 stem. Moreover, the average area of F_3_1 abaxial epidermal pavement cells from the distal lamina sector of the most expanded leaves was increased compared to wt congruently with an effect of *GFP*-*CesA6* expression on cell expansion. Moreover, in F_3_1 leaves, we also observed the overexpression of *PIP1* and *PIP2*; these genes code for two aquaporin isoforms assembling in heterotrimers at the plasma membrane and able to mediate significant transmembrane water fluxes [71]. *PIP1* and *PIP2* were reported to be involved in the expansion of rose petals [72] and pomegranate flowers [73], so that the overexpression of these two *PIPs* in F_3_1 line can be considered a further indication on the effect of *GFP*-*CesA6* expression on the expansion growth.

## 5. Conclusions

Not all attempts to increase the biosynthesis of cellulose through the overexpression of the *CesA* genes have given positive results. In transgenic barley lines, overexpressing primary and secondary *CesAs*, no increase in the cellulose content was obtained and, in some lines, it decreased significantly. A decrease of cell wall thickness, dwarf plants, and a “brittle node” phenotype were also found and justified as the need for a coordinated overexpression of all three *HvCesA* genes for the formation of a functional complex [20]. Taken together, the results presented here highlight that the *AtCesA6* GFP-tagged expression led to the formation of tobacco plants with a “giant” phenotype with an evident increase of plant biomass. *AtCesA6* affected cell wall biosynthetic machinery involved in lignocellulosic biomass production: (i) enhancing the expression of other tobacco primary wall *CesAs* (*NtCesA1*, *NtCesA3*, and *NtCesA6*) to produce more cellulose and those of other genes involved in the cell wall construction and *modification* (*NtXyl4*, *NtGal10*, *NtExp11*, *NtPIP1.1*, and *NtPIP2.7*); (ii) causing the relative amounts of cell wall polysaccharides and possibly of non-polysaccharide material in transformed tobacco seedlings; and (iii) increasing secondary cell wall deposition clearly visible in tobacco secondary growth of the stem. We prospect the possibility of using tobacco plants expressing the exogenous gene *GFP*-*CesA6* to increase plant biomass for sustainable development and the diversification of the energy sources.

## Figures and Tables

**Table 1:** Average values of parameters of wt and F_3_1 tobacco flowers and seeds; the data are the mean ± SD of 10 measurements on each line. Asterisk indicates significant differences (*p* < 0.05; *), according to the *t*-Student test, between the two samples.

	wt	F_3_1
Anthesis	28 weeks ± 5 days	25 weeks ± 3 days *
Flower numbers	9 ± 1	13 ± 2 *
Seed numbers	9659 ± 147	11.516 ± 250 *
Seed total weight (g)	0.749 ± 0.047	0.979 ± 0.093 *

**Table 2:** Chlorophyll and carotenoid content in wt and F_3_1 tobacco leaves. The data represent the mean ± SD of four independent experiments. The asterisks indicate significant (*p* < 0.05; *) or highly significant (*p* < 0.001, **) differences, according to the t-Student test, between the two samples.

	nmol/g fw					
	Chl a	Chl b	Chl (a+b)	Car	Chla:Chlb	Chl (a+b):Car
wt	143.1 ± 2.0	38.8 ± 2.9	181.9 ± 4.8	51.6 ± 2.5	3.7	3.53
F_3_1	123.9 ± 9.1 **	31.7 ± 8.5	155.6 ± 13.6 *	54.5 ± 3.7	3.9	2.86

**Table 3:** Weights of the purified cell walls and extracts obtained from the leaves of wt and F_3_1 2-month-old tobacco seedlings. The data, expressed in mg/g fw, represent the mean ± standard deviation of four independent experiments. Percentage values refer to the sum of total weight of extracts. In parenthesis are reported the amounts of cellulose quantified in the insoluble residues by the Updegraff method [36]. Asterisks indicate significant (*p* < 0.05; *) or highly significant (*p* < 0.001 **) differences, according to the *t*-Student test, between wt and F_3_1 samples. FC, fold change.

	wt		F_3_1		*p*-Value	FC
	mg/g fw		mg/g fw			
Purified cell wall	7.3 ± 0.6		17.6 ± 1.3 **		<0.001	2.41
Extracts	mg/g fw	%	mg/g fw	%	*p*-value	FC
CDTA + Na_2_CO_3_	1.59 ± 0.08	22.8	1.75 ± 0.10 *	9.9	0.047	1.10
0.5–4.0 M KOH	0.74 ± 0.07	10.6	0.91 ± 0.10 *	5.2	0.032	1.23
Insoluble residue (of which cellulose)	4.63 ± 0.87 (1.27 ± 0.14)	66.5 (18.2)	15.0 ± 2.28 ** (4.18 ± 0.56 **)	84.9 (23.7)	<0.001 (<0.001)	3.24 (3.29)

**Table 4:** Percentage distribution of pectins, hemicelluloses, and cellulose in the purified cell walls from the leaves of wt and F_3_1 2-month-old tobacco seedlings. The percentage values refer to the total weight of the polysaccharide material expressed in mg/g of dry weight (dw) of purified cell walls and represent the mean of four independent experiments. The asterisk (*) indicates significant (*p* < 0.05) difference, according to the *t*-Student test, between wt and F_3_1 samples.

	Relative Percent (%)	
	wt	F_3_1
Pectins	44.2	25.6
Hemicelluloses	20.6	13.3
Cellulose	35.2	61.1
Total polysaccharides (mg/g dw)	493.2 ± 41.4	388.6 ± 42.7 *

## Data Availability

The data presented in this study are available upon request from the corresponding authors.

## Supplementary Materials

The following supporting information can be downloaded at: https://www.mdpi.com/article/10.3390/biology11081139/s1, Figure S1. Western blot membrane of membrane protein fractions in leaves of 2-month-old wt and transformed tobacco seedlings stably expressing GFP-CesA6; bands were detected by anti-GFP (1:5000 *v*/*v*) (Molecular Probes, now ThermoFisher Scientific). Weight marker (molecular weight in kDa): Biorad Prestained SDS-PAGE Standards, broad range (7100–209,000 MW). Densitometry readings/intensity ratio of each band on Western Blot. SD: Standard Deviation; SE: Standard Error. Figure S2. Western blot of soluble (SOL), membrane (MEM), and cell wall (CW) proteins of wt and F_3_1 tobacco leaves of 2-month-old seedlings. XTH abundance in the cell wall fraction protein of wt and F_3_1 lines. Bands were detected by anti-XTH (Agrisera). Weight marker (molecular weight in kDa): Precision Plus Protein™ All Blue Prestained Protein Standards, Biorad, mixture of ten blue-stained recombinant proteins (10–250 kD), including three reference bands (25, 50, 75 kD). Densitometry readings/intensity ratio of each band on Western Blot. SD: Standard Deviation; SE: Standard Error. Figure S3: Stacked plot of representative ^1^H-NMR (600 MHz) spectra expanded area, in the range of (a) 2–3.5 ppm and (b) 4–7.5 ppm, for F_3_1 and wt CDTA + Na_2_CO_3_ hydrolyzates (pectins). Assignment of selected components is indicated. GalA = galacturonic acid; OGAs = Oligogalacturonides; Rha = rhamnose; MeO = Methoxy groups; Ac = Acetyl groups; FCA = ferulic acid; Figure S4: Stacked plot of representative ^1^H-NMR (600 MHz) spectra expanded area (4.4–5.50 ppm) for F_3_1 and wt KOH extracts (hemicelluloses). Assignment of selected components is indicated. Man = mannose; Glc = glucose; Xyl = xylose; Fuc = fucose; Figure S5: Thin layer chromatography (TLC) of analysis of the hydrolyzates from CDTA + Na_2_CO_3_ (pectins) and KOH 0.5–4.0 M (hemicelluloses) extracts obtained from the purified cell walls from the leaves of wt and F_3_1 tobacco 2-month-old seedlings. Rhamnose (Rha), xylose (Xyl), arabinose (Ara), mannose (Man), glucose (Glc), galactose (Gal), and acid galacturonic (GalA) were used as authentic standards (Std); Table S1: Primer for gene expression analysis; Table S2: Tested reference genes.

## References

[1] Cosgrove D.J., Jarvis M.C. (2012). Comparative structure and biomechanics of plant primary and secondary cell walls. Front. Plant Sci..

[2] Brown R.M. (2004). Cellulose structure and biosynthesis: What is in store for the 21st century?. J. Polym. Sci. Part A Polym. Chem..

[3] Desprez T., Juraniec M., Crowell E.F., Jouy H., Pochylova Z., Parcy F., Höfte H., Gonneau M., Vernetthes S. (2007). Organization of cellulose synthase complexes involved in primary cell wall synthesis in *Arabidopsis thaliana*. Proc. Natl. Acad. Sci. USA.

[4] Polko J.K., Kieber J.J. (2019). The regulation of cellulose biosynthesis in plants. Plant Cell.

[5] Rodriguez-Restrepo Y.A., Rocha C.M.R., Teixeira J.A., Orrego C.E. (2020). Valorization of Passion Fruit Stalk by the Preparation of Cellulose Nanofibers and Immobilization of Trypsin. Fibers. Polym..

[6] Herth W. (1983). Arrays of plasma-membrane “rosettes” involved in cellulose microfibril formation of Spirogyra. Planta.

[7] Doblin M., Kurek I., Jacob-Wilk D., Delmer D.P. (2002). Cellulose biosynthesis in plants: From genes to rosettes. Plant Cell Physiol..

[8] Somerville C. (2006). Cellulose synthesis in higher plants. Annu. Rev. Cell Dev. Biol..

[9] Guerriero G., Fugelstad J., Bulone V. (2010). What do we really know about cellulose biosynthesis in higher plants?. J. Integr. Plant Biol..

[10] Newman R.H., Hill S.J., Harris P.J. (2013). Wide-angle x-ray scattering and solid-state nuclear magnetic resonance data combined to test models for cellulose microfibrils in mung bean cell walls. Plant Physiol..

[11] Zhang T., Zheng Y., Cosgrove D.J. (2016). Spatial organization of cellulose microfibrils and matrix polysaccharides in primary plant cell walls as imaged by multichannel atomic force microscopy. Plant J..

[12] Purushotham P., Ho R., Zimmer J. (2020). Architecture of a catalytically active homotrimeric plant cellulose synthase complex. Science.

[13] Duncombe S.G., Chethan S.G., Anderson C.T. (2022). Super-resolution imaging illuminates new dynamic behaviors of cellulose synthase. Plant Cell.

[14] Gonneau M., Desprez T., Guillot A., Vernhettes S., Höfte H. (2014). Catalytic subunit stoichiometry within the cellulose synthase complex. Plant Physiol..

[15] Hill J.L., Hammudi M.B., Tien M. (2014). The Arabidopsis cellulose synthase complex: A proposed hexamer of CESA trimers in an equimolar stoichiometry. Plant Cell.

[16] Purushotham P., Cho S.H., Díaz-Moreno S.M., Kumar M., Nixon B.T., Bulone V., Zimmer J. (2016). A single heterologously expressed plant cellulose synthase isoform is sufficient for cellulose microfibril formation in vitro. Proc. Natl. Acad. Sci. USA.

[17] Cho S.H., Purushotham P., Fang C., Maranas C., Díaz-Moreno S.M., Bulone V., Zimmer J., Kumar M., Nixon B.T. (2017). Synthesis and self-assembly of cellulose microfibrils from reconstituted cellulose synthase. Plant Physiol..

[18] Zhang X., Dominguez P.G., Kumar M., Bygdell J., Miroshnichenko S., Sundberg B., Wingsle G., Niittylä T. (2018). Cellulose synthase stoichiometry in aspen differs from Arabidopsis and Norway spruce. Plant Physiol..

[19] Joshi C.P., Thammannagowda S., Fujino T., Gou J.Q., Avci U., Haigler C.H., McDonnell L.M., Mansfield S.D., Mengesha B., Carpita N.C. (2011). Perturbation of wood cellulose synthesis causes pleiotropic effects in transgenic Aspen. Mol. Plant.

[20] Tan H., Shirley N.J., Singh R.R., Henderson M., Dhugga K.S., Mayo G.M., Fincher G.B., Burton R.A. (2015). Powerful regulatory systems and posttranscriptional gene silencing resist increases in cellulose content in cell walls of barley. BMC Plant Biol..

[21] Mazarei M., Baxter H.L., Li M., Biswal A.K., Kim K., Meng X., Pu Y., Wuddineh W.A., Zhang J.-Y., Turner G.B. (2018). Functional Analysis of Cellulose Synthase CesA4 and CesA6 Genes in Switchgrass (*Panicum virgatum*) by Overexpression and RNAi-Mediated Gene Silencing. Front. Plant Sci..

[22] Hu H.Z., Zhang R., Feng S.Q., Wang Y.M., Wang Y.T., Fan C.F., Li Y., Liu Z., Schneider R., Xia T. (2018). Three AtCesA6-like members enhance biomass production by distinctively promoting cell growth in Arabidopsis. Plant Biotechnol. J..

[23] De Caroli M., Manno E., Perrotta C., De Lorenzo G., Di Sansebastiano G.-P., Piro G. (2020). CesA6 and PGIP2 Endocytosis Involves Different Subpopulations of TGN-Related Endosomes. Front Plant Sci..

[24] Gutierrez R., Lindeboom J.J., Paredez A.R., Emons A.M.C., Ehrhardt D.W. (2009). Arabidopsis cortical microtubules position cellulose synthase delivery to the plasma membrane and interact with cellulose synthase trafficking compartments. Nat. Cell Biol..

[25] Crowell E.F., Gonneau M., Stierhof Y.D., Höfte H., Vernhettes S. (2010). Regulated trafficking of cellulose synthases. Curr. Opin. Plant Biol..

[26] Li S., Li L., Fan W., Ma S., Zhang C., Kim J.C., Wang K., Russinova E., Zhu Y., Zhou Y. (2022). LeafNet: A tool for segmenting and quantifying stomata and pavement cells. Plant Cell.

[27] Ursache R., Andersen T.G., Marhavý P., Geldner N. (2018). A protocol for combining fluorescent proteins with histological stains for diverse cell wall components. Plant J..

[28] Lichtenthaler H.K., Buschmann C. (2001). Chlorophylls and Carotenoids: Measurement and Characterization by UV-VIS Spectroscopy. Curr. Prot. Food Analyt. Chem..

[29] De Caroli M., Manno E., Piro G., Lenucci M.S. (2021). Ride to cell wall: Arabidopsis XTH11, XTH29 and XTH33 exhibit different secretion pathways and responses to heat and drought stress. Plant J..

[30] Schmidt G.W., Delaney S.K. (2010). Stable internal reference genes for normalization of real-time RT-PCR in tobacco (*Nicotiana tabacum*) during development and abiotic stress. Mol. Genet. Genom..

[31] Czechowski T., Stitt M., Altmann T., Udvardi M.K., Scheible W.-R. (2005). Genome-Wide Identification and Testing of Superior Reference Genes for Transcript Normalization in *Arabidopsis*. Plant Physiol..

[32] Chen J.J., Wang S.-J., Tsai C.-A., Lin C.-J. (2007). Selection of differentially expressed genes in microarray data analysis. Pharm. J..

[33] Bradford M.M. (1976). A rapid and sensitive method for the quantitation of microgram quantities of protein utilizing the principle of protein-dye binding. Analyt. Biochem..

[34] Leucci M.R., Lenucci M.S., Piro G., Dalessandro G. (2008). Water stress and cell wall polysaccharides in the apical root zone of wheat cultivars varying in drought tolerance. J. Plant Physiol..

[35] Lenucci M.S., Durante M., Anna M., Dalessandro G., Piro G. (2013). Possible use of the carbohydrates present in tomato pomace and in byproducts of the supercritical carbon dioxide lycopene extraction process as biomass for bioethanol production. J. Agric. Food Chem..

[36] Updegraff D.M. (1969). Semimicro determination of cellulose in biological materials. Analytic. Biochem..

[37] Burana-osot J., Soonthornchareonnon N., Hosoyama S., Linhardt R.J., Toida T. (2010). Partial depolymerization of pectin by a photochemical reaction. Carbohydr. Res..

[38] Merkx D.W.H., Westphal Y., van Velzen E.J.J., Thakoer K.V., de Roo N., van Duynhoven J.P.M. (2018). Quantification of food polysaccharide mixtures by 1H NMR. Carbohydr. Polym..

[39] Zhi Z., Chen J., Li S., Wang W., Huang R., Liu D., Ding T., Linhardt R.J., Chen S., Ye X. (2017). Fast preparation of RG-I enriched ultra-low molecular weight pectin by an ultrasound accelerated Fenton process. Sci. Rep..

[40] Thöle C., Brandt S., Ahmed N., Hensel A. (2015). Acetylated rhamnogalacturonans from immature fruits of *Abelmoschus esculentus* inhibit the adhesion of *Helicobacter pylori* to human gastric cells by interaction with outer membrane proteins. Molecules.

[41] Shi H., Yu L., Shi Y., Lu J., Teng H., Zhou Y., Sun L. (2017). Structural Characterization of a Rhamnogalacturonan I Domain from Ginseng and Its Inhibitory Effect on Galectin-3. Molecules.

[42] Milliasseau D., Jeftić J., Pessel F., Plusquellec D., Benvegnu T. (2021). Transformation of Pectins into Non-Ionic or Anionic Surfactants Using a One-Pot and Cascade Mode Process. Molecules.

[43] Wishart D.S. (2008). Quantitative metabolomics using NMR, TrAC. Trends Analyt. Chem..

[44] Girelli C.R., De Pascali S.A., Del Coco L., Fanizzi F.P. (2016). Metabolic profile comparison of fruit juice from certified sweet cherry trees (*Prunus avium* L.) of Ferrovia and Giorgia cultivars: A preliminary study. Food Res. Internat..

[45] Del Coco L., Felline S., Girelli C.R., Angilè F., Magliozzi L., Almada F., D’Aniello B., Mollo E., Terlizzi A., Fanizzi F.P. (2018). 1H NMR Spectroscopy and MVA to Evaluate the Effects of Caulerpin-Based Diet on Diplodus sargus Lipid Profiles. Mar. Drugs.

[46] O’Rourke C., Gregson T., Murray L., Sadler I.H., Fry S.C. (2015). Sugar composition of the pectic polysaccharides of charophytes, the closest algal relatives of land-plants: Presence of 3-O-methyl-D-galactose residues. Annals Bot..

[47] De Caroli M., Lenucci M.S., Manualdi F., Dalessandro G., De Lorenzo G., Piro G. (2015). Molecular dissection of Phaseolus vulgaris polygalacturonase-inhibiting protein 2 reveals the presence of hold/release domains affecting protein trafficking toward the cell wall. Front. Plant Sci..

[48] Leucci M.R., Di Sansebastiano G.P., Gigante M., Dalessandro G., Piro G. (2007). Secretion marker proteins and cell-wall polysaccharides move through different secretory pathway. Planta.

[49] Schmittgen T.D., Livak K.J. (2008). Analyzing real-time PCR data by the comparative C(T) method. Nat. Protoc..

[50] De Rosa A., Watson-Lazowski A., Evans R.J., Groszmann M. (2020). Genome-wide identification and characterisation of Aquaporins in *Nicotiana tabacum* and their relationships with other *Solanaceae* species. BMC Plant Biol..

[51] Nishitani K., Vissenberg K., Verbelen J.P., Vissenberg K. (2006). Roles of the XTH protein family in the expanding cell. The Expanding Cell.

[52] Garna H., Mabon N., Nott K., Wathelet B., Paquot M. (2006). Kinetic of the hydrolysis of pectin galacturonic acid chains and quantification by ionic chromatography. Food Chem..

[53] Mohnen D. (2008). Pectin structure and biosynthesis. Curr. Opin. Plant Biol..

[54] Fry S.C. (1986). Cross-linking of matrix polymers in the growing cell walls of angiosperms. Annu. Rev. Plant Physiol..

[55] Carroll A., Somerville C. (2009). Cellulosic biofuels. Annu. Rev. Plant Biol..

[56] Lenucci M.S., De Caroli M., Marrese P.P., Iurlaro A., Rescio L., Volker B., Dalessandro G., Piro G. (2015). Enzyme-aided extraction of lycopene from high-pigment tomato cultivars by supercritical carbon dioxide. Food Chem..

[57] Li F., Xie G., Huang J., Zhang R., Li Y., Zhang M., Wang Y., Li A., Li X., Xia T. (2017). OsCESA9 conserved-site mutation leads to largely enhanced plant lodging resistance and biomass enzymatic saccharification by reducing cellulose DP and crystallinity in rice. Plant Biotechnol. J..

[58] Wang Y., Fan C., Hu H., Li Y., Sun D., Peng L. (2016). Genetic modification of plant cell walls to enhance biomass yield and biofuel production in bioenergy crops. Biotechnol. Adv..

[59] Maleki S.S., Mohammadi K., Movahedi A., Wu F., Ji K.S. (2020). Increase in cell wall thickening and biomass production by overexpression of PmCesA2 in poplar. Front. Plant Sci..

[60] Li Y., He N., Hou J., Xu L., Liu C., Zhang J., Wang Q., Zhang X., Wu X. (2018). Factors Influencing Leaf Chlorophyll Content in Natural Forests at the Biome Scale. Front. Ecol. Evol..

[61] Bhattacharyya D., Gnanasekaran P., Kumar R.K., Kushwaha N.K., Sharma V.K., Yusuf M.A., Chakraborty S. (2015). A geminivirus betasatellite damages the structural and functional integrity of chloroplasts leading to symptom formation and inhibition of photosynthesis. J. Exp. Bot..

[62] Lichtenthaler H.K., Babani F., Papageorgiou G.C., Govindjee (2004). Light adaptation and senescence of the photosynthetic apparatus. Changes in pigment composition, chlorophyll fluorescence parameters and photosynthetic activity. Chlorophyll a Fluorescence a Signature of Photosynthesis.

[63] Ferroni L., Suorsa M., Aro E.-M., Baldisserotto C., Pancaldi S. (2016). Light acclimation in the lycophyte *Selaginella martensii* depends on changes in the amount of photosystems and on the flexibility of the light-harvesting complex II antenna association with both photosystems. New Phytol..

[64] Lichtenthaler H.K., Schweiger J. (1998). Cell wall bound ferulic acid, the major substance of the blue-green fluorescence emission of plants. J. Plant Physiol..

[65] Carroll A., Mansoori N., Li S., Lei L., Vernhettes S., Visser R.G.F., Somerville C., Gu Y., Trindade L.M. (2012). Complexes with mixed primary and secondary cellulose synthases are functional in *Arabidopsis* plants. Plant Physiol..

[66] Gu F., Bringmann M., Combs J.R., Yang J., Bergmann D.C., Nielsen E. (2016). *Arabidopsis* CSLD5 functions in cell plate formation in a cell cycle-dependent manner. Plant Cell..

[67] Yang W., Schuster C., Beahan C.T., Charoensawan V., Peaucelle A., Bacic A., Doblin M.S., Wightman R., Meyerowitz E.M. (2016). Regulation of meristem morphogenesis by cell wall synthases in *Arabidopsis*. Curr. Biol..

[68] Hunter C.T., Kirienko D.H., Sylvester A.W., Peter G.F., McCarty D.R., Koch K.E. (2012). Cellulose synthase-like D1 is integral to normal cell division, expansion, and leaf development in maize. Plant Physiol..

[69] Xi H., Liu J., Li Q., Chen X., Liu C., Zhao Y., Yao J., Chen D., Si J., Liu C. (2021). Genome-wide identification of Cellulose-like synthase D gene family in *Dendrobium catenatum*. Biotechn. Biotechn. Equip..

[70] Yoshikawa T., Eiguchi M., Hibara K., Ito J., Nagato Y. (2013). Rice slender leaf 1 gene encodes cellulose synthase-like D4 and is specifically expressed in M-phase cells to regulate cell proliferation. J. Exp. Bot..

[71] Bienert M.D., Diehn T.A., Richet N., Chaumont F., Bienert G.P. (2018). Heterotetramerization of plant PIP1 and PIP2 aquaporins is an evolutionary ancient feature to guide PIP1 plasma membrane localization and function. Front. Plant Sci..

[72] Chen W., Yin X., Wang L., Tian J., Yang R., Liu D., Yu Z., Ma N., Gao J. (2013). Involvement of rose aquaporin RhPIP1;1 in ethylene-regulated petal expansion through interaction with RhPIP2;1. Plant Mol. Biol..

[73] Liu J., Qin G., Liu C., Liu X., Zhou J., Li J., Lu B., Zhao J. (2021). Genome-wide identification of candidate aquaporins involved in water accumulation of pomegranate outer seed coat. Peer J..

