# Supplementary material for: Expression of Exogenous GFP-CesA6 in Tobacco Enhances Cell Wall Biosynthesis and Biomass Production
Source: Biology (Basel). 2022 Jul 29;11(8):1139. doi: 10.3390/biology11081139 (PMC9405164; doi:10.3390/biology11081139)
Supplement: Supplementary file 1 [file biology-11-01139-s001.zip › biology-1813354-supplementary.pdf]

biology

MDPI

Article

Expression of Exogenous *GFP-CesA6* in Tobacco Enhances Cell Wall Biosynthesis and Biomass Production

Monica De Caroli, Patrizia Rampino, Gabriele Pecatelli, Chiara Roberta Girelli, Francesco Paolo Fanizzi, Gabriella Piro and Marcello S. Lenucci

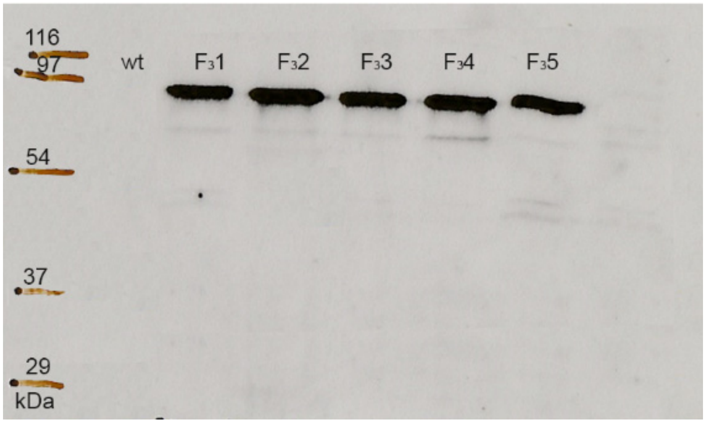

Experiment 1 (R1)

wt	0	0
F_31	121,4375	98,13131
F_32	123,3125	99,64646
F_33	121,9375	98,53535
F_34	122,4688	98,96465
F_35	123,75	100

Experiment 2 (R2)

wt	0	0
F_31	122,751	100,1538
F_32	122,4375	99,89801
F_33	125,125	102,0908
F_34	123,6875	100,9179
F_35	122,5625	100

Experiment 3 (R3)

wt	0	0
F_31	124,251	102,2133
F_32	124,8438	102,7009
F_33	123,3438	101,467
F_34	124,8125	102,6752
F_35	121,5605	100

	CI	C2	C3	Media	dev	err	Significance t test
wt	0	0	0	0	0	0	0
F31	98,13131	100,1538	102,2133	100,1661	1,267517	0,89627	0,812787
F32	99,64646	99,89801	102,7009	100,7485	1,610079	1,138498	0,497199
F33	98,53535	102,0908	101,467	100,6977	1,68135	1,188894	0,560563
F34	98,96465	100,9179	102,6752	100,8526	1,2634	0,893359	0,492731
F35	100	100	100	100	0	0	0

Figure S1. Western blot membrane of membrane protein fractions in leaves of 2-month-old wt and transformed tobacco seedlings stably expressing GFP-CesA6; bands were detected by anti-GFP (1:5000 v/v) (Molecular Probes, now ThermoFisher Scientific). Weight marker (molecular weight in kDa): Biorad Prestained SDS-PAGE Standards, broad range (7100–209,000 MW). Densitometry readings/intensity ratio of each band on Western Blot. SD: Standard Deviation; SE: Standard Error.

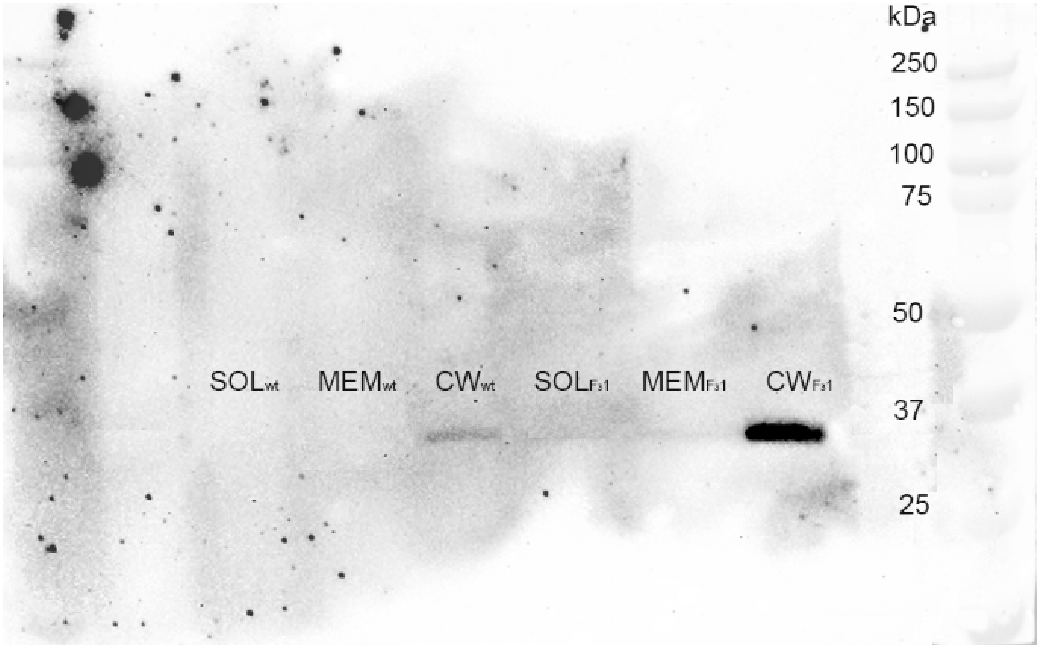

wt1	76,697	47,52424
F_31_1	161,385	100
wt2	69,557	47,40443
F_31_2	146,731	100
wt3	66,049	47,84461
F_31_3	138,049	100

							Significance
	R1	R2	R3	average	SD	SE	t-test
wt	47,52424	47,40443	47,84461	47,59109	0,227573	0,160919	2,37004E-10
F_31	100	100	100	100	0	0	0

Figure S2. Western blot of soluble (SOL), membrane (MEM) and cell wall (CW) proteins of wt and F_31 tobacco leaves of 2-month-old seedlings. XTH abundance in the cell wall fraction protein of wt and F_31 lines. Bands were detected by anti-XTH (Agrisera). Weight marker (molecular weight in kDa): Precision Plus Protein™ All Blue Prestained Protein Standards, Biorad, mixture of ten blue-stained recombinant proteins (10–250 kD), including three reference bands (25, 50, 75 kD). Densitometry readings/intensity ratio of each band on Western Blot. SD: Standard Deviation; SE: Standard Error.

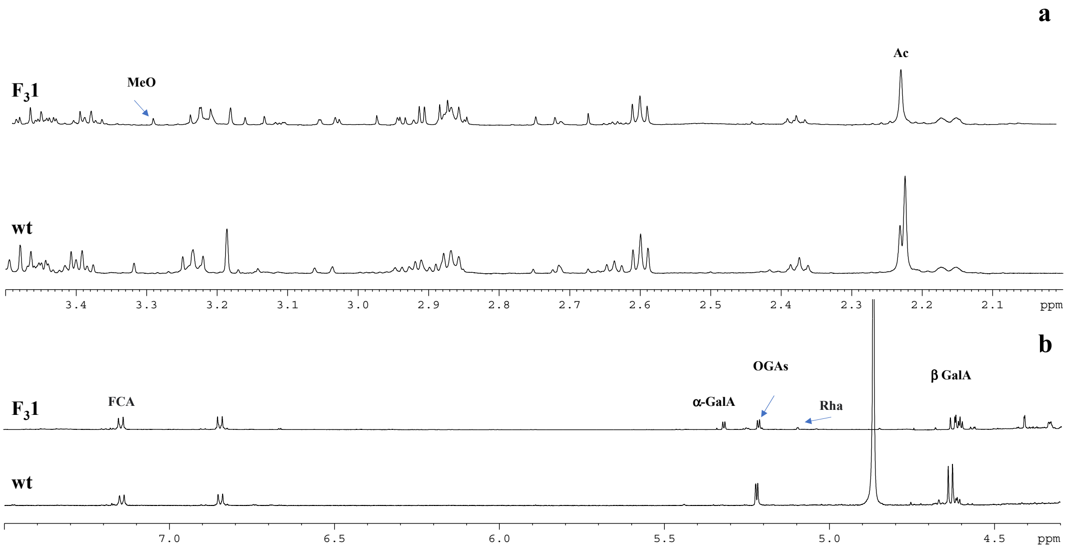

Figure S3. Stacked plot of representative ^{1}H-NMR (600 MHz) spectra expanded area, in the range of (**a**) 2–3.5 ppm and (**b**) 4–7.5 ppm, for F31 and wt CDTA + Na_2CO_3 hydrolysates (pectins). Assignment of selected components is indicated. GalA = galacturonic acid; OGAs = Oligogalacturonides; Rha = rhamnose; MeO = Methoxy groups; Ac = Acetyl groups; FCA = ferulic acid.

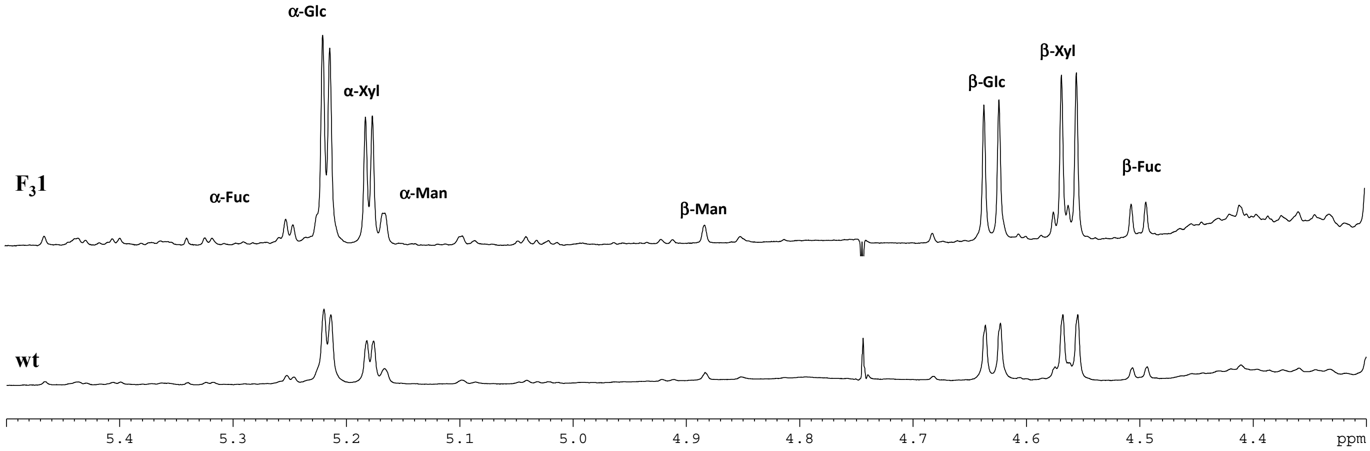

Figure S4. Stacked plot of representative ^{1}H-NMR (600 MHz) spectra expanded area (4.4-5.50 ppm) for F31 and wt KOH extracts (hemicelluloses). Assignment of selected components is indicated. Man = mannose; Glc = glucose; Xyl = xylose; Fuc = fucose.

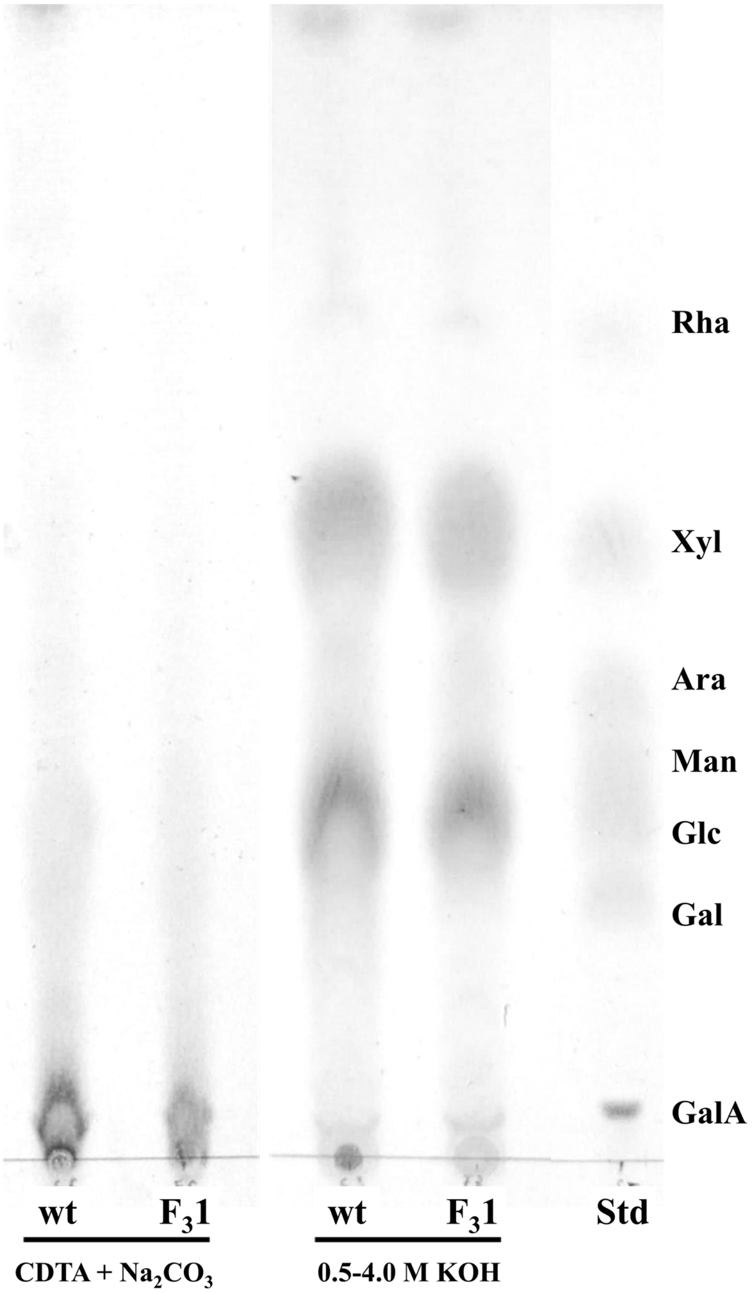

Figure S5. Thin layer chromatography (TLC) of analysis of the hydrolysates from CDTA + Na_2CO_3 (pectins) and KOH 0,5-4,0M (hemicelluloses) extracts obtained from the purified cell walls from the leaves of wt and $F_3$1 tobacco 2-month-old seedlings. Rhamnose (Rha), xylose (Xyl), arabinose (Ara), mannose (Man), glucose (Glc), galactose (Gal) and acid galacturonic (GalA) were used as authentic standards (Std).

Table S1. Primer for gene expression analysis.

NtCesA1for	5′-TGCCAGATTTGTGGAGATGA-3′
NtCesA1rev	5′-TCATTGCAAGCGATAAATGG-3′
NtCesA3for	5′-AAGTGGAGGGCTTGACAGTGAT-3′
NtCesA3rev	5′-GACACTCGAACCAGTGCATTCA-3′
NtCesA6for	5′-GAGTCTCTGCAGTGCAACCA-3′
NtCesA6rev	5′-GATTGCCTTCTCGTCTTTCG-3′
NtCesA4for	5′-CGGCTATGATCCACCTGTTT-3′
NtCesA4rev	5′-TCTTGAACCACCACAACAGC-3′
NtCesA7for	5′-CCGTGGTTGGAGATCAGTTT-3′
NtCesA7rev	5′-AAGCACCTGGTTGAGACGAT-3′
NtCesA8for	5′-GTCTCTGCCCATCTCTTTGC-3′
NtCesA8rev	5′-AGAGATCGCCAAACTCTCCA-3′
NtXyl4for	5′-GATTTCCAACCGAATCCTGA-3′
NtXyl4rev	5′-TAGTCCAACATCCGGCTTTC-3′
NtGal10for	5′-GGTGAACTACGCTGCAATGA-3′
NtGal10rev	5′-CCTCGGAAACTGTTCATGGT-3′
NtExp11for	5′-GGTGTAACCCTCCTCGACAA-3′
NtExp11rev	5′-CCACCTCTGTAAACGCCAAT-3′
NtPIP1.1for	5′-TCTACTCCTTGCCGTGTTCC-3′
NtPIP1.1rev	5′-AGATGACCGGATTGAGATGG-3′
NtPIP2.7for	5′-CGGCGGAGTATTCTGCTAAG-3′
NtPIP2.7rev	5′-AATAAGCGGAGTTGGTGGTG-3′
NtEF1Afor	5′-TGAGATGCACCACGAAGCTC-3′
NtEF1Arev	5′-CCAACATTGTCACCAGGAAGTG-3′
NtL25for	5′-AAGGGTGTTGTTGTCCTCAATCTT-3′
NtL25rev	5′-AAGGGTGTTGTTGTCCTCAATCTT-3′
NtNtubc2for	5′-CTGGACAGCAGACTGACATC-3′
NtNtubc2rev	5′-CAGGATAATTTGCTGTAACAGATTA-3′
NtTubA1for	5′-CAAGACTAAGCGTACCATCCA-3′
NtTubA1rev	5′-TTGAATCCAGTAGGGCACCAG-3′

Table S2. Tested reference genes.

	**NtEF1A**	**SD**	**NtL25**	**SD**	**NtNtubC2**	**SD**	**NtTubA1**	**SD**
wt leaf cyclethreshold	23,24	0,478218	24,5	0,747707	26,03	0,659436	23,42	1,391639
	24,03	**average**	24,49	**average**	26,05	**average**	27,34	**average**
	23,83	23,01409	26,59	24,25909	26,3	25,73227	26,74	23,81864
	23,39	**CV**	24,24	**CV**	25,09	**CV**	22,5	**CV**
	23,26	**0,020779**	24,28	**0,030822**	26,21	**0,025627**	24,36	**0,058426**
	23,43		24,39		26,03		23,31	
	22,93		24,18		24,89		22,38	
	23,01		24		25,5		22,49	
	23,01		24,24		25,94		22,92	
	23,17		24,54		26,56		26,01	
	23,43		25,32		25,97		25,1	
$F_3$1 leaf cyclethreshold	22,67		24,09		25,06		22,92	
	23,19		24,33		25,3		24,13	
	23,04		24,26		26,1		23,15	
	22,22		22,41		24,84		22,19	
	22,25		23,82		25,83		22,5	
	22,3		23,66		25,66		23,33	
	22,91		23,49		25		23,56	
	23,11		24,02		25,34		23,87	
	22,91		24,4		25,41		23,99	
	22,39		24,56		27,68		24,13	
	22,59		23,89		25,32		23,67	
wt stem cyclethreshold	21,23	**SD**	25,1	**SD**	26,13	**SD**	22,78	**SD**
	22,65	1,025214	24,9	1,440836	25,98	1,771673	24,89	1,600263
	24,57	**average**	24,12	**average**	22,76	**average**	25,98	average
	24,87	23,375	23,97	24,57917	26,35	25,68583	22,67	24,27667
	23,26	**CV**	24,34	**CV**	27,04	**CV**	25,54	**CV**
	23,57	**0,043859**	25,89	**0,05862**	26,89	**0,068975**	22,56	**0,065918**
$F_3$1 stem cyclethreshold	23,3		24,22		22,67		23,65	
	24,01		26,98		25,89		24,12	
	24,28		22,99		24,88		25,37	
	23,57		25,34		27,43		27,45	
	22,25		21,43		28,2		23,97	
	22,94		25,67		24,01		22,34	
